# Cardiovascular and Clinical Manifestations of Marfan Syndrome and Other Inherited Connective Tissue Disorders with Coexisting Genetic Variants

**DOI:** 10.3390/cells15111001

**Published:** 2026-05-29

**Authors:** Maria Elena Soto, Gilberto Vargas-Alarcón, Claudia Huesca-Gómez, Israel Pérez-Torres, José Antonio Arias-Godínez, Sergio Enrique Meza-Toledo, Regina de la Mora-Cervantes, Hugo Rodríguez-Zanella, Gabriela Meléndez-Ramírez, Linaloe Manzano-Pech, Giovanny Fuentevilla-Álvarez, Ricardo Gamboa

**Affiliations:** 1 Research Direction, Instituto Nacional de Cardiología Ignacio Chávez, Juan Badiano No. 1. Col. Sección XVI, México City 14080, Mexico; gvargas63@yahoo.com; 2 Cardiovascular Line in American British Cowdray (ABC) Medical Center, PAI ABC Sur 136 No. 116, Col. Las America’s, México City 01120, Mexico; 3 Physiology Department, Instituto Nacional de Cardiología Ignacio Chávez, Juan Badiano No. 1. Col. Sección XVI, México City 14080, Mexico; c_huesca@yahoo.com; 4 Cardiovascular Biomedicine Department, Instituto Nacional de Cardiología Ignacio Chávez, Juan Ba-diano No. 1. Col. Sección XVI, México City 14080, Mexico; pertorisr@yahoo.com.mx (I.P.-T.);; 5 Echocardiography Department, Instituto Nacional de Cardiologia Ignacio Chávez Juan Badiano No. 1. Col. Sección XVI, México City 14080, Mexico; jaag1407@yahoo.com (J.A.A.-G.); drzanella@gmail.com (H.R.-Z.); 6 Biochemistry Department, Escuela Nacional de Ciencias Biológicas, Instituto Politécnico Nacional (IPN), Manuel Carpio y Plutarco Elias Calles, Col. Miguel Hidalgo, México City 11350, Mexico; semeza@hotmail.com; 7 Computed Tomography Instituto Nacional de Cardiología Ignacio Chávez, Juan Badiano No. 1. Col. Sección XVI, México City 14080, Mexico; reginadelamora@hotmail.com; 8 Magnetic Resonance Department, Instituto Nacional de Cardiologia Ignacio Chávez Juan Badiano No. 1. Col. Sección XVI, México City 14080, Mexico; gabrielamelram@yahoo.com.mx; 9 Endocrinology Department, Instituto Nacional de Cardiología Ignacio Chávez, Juan Badiano No. 1. Col. Sección XVI, México City 14080, Mexico

**Keywords:** genetic variant genes of sarcomere cardiomyopathies, arrhythmias, structural abnormalities and hypercholesterolemia, Marfan syndrome, Loeys–Dietz syndrome, Beals–Hecht syndrome, Ehlers–Danlos syndrome, undifferentiated connective tissue disease

## Abstract

**Highlights:**

**What are the main findings?**

- The exploratory study provided preliminary results on the co-occurrence of pathogenic variants in genes other than those associated with each syndrome.
- The expanded and exploratory genetic analysis using next-generation sequencing provided preliminary results on the coexistence of pathogenic variants in genes other than those associated with each syndrome and was linked to severe and heterogeneous cardiovascular and musculoskeletal clinical phenotypes.

**What are the implications of the main findings?**

- Marfan syndrome (MS) and other syndromes are associated with a high cardiovascular risk, which can occur in isolation or in combination with aneurysms, aortic dissection, valvular prolapse, cardiac dysfunction, heart failure, and arrhythmias. Although patients present with distinctive clinical features, diagnosis is often delayed due to insufficient information at the initial consultation.
- These findings highlight the need to incorporate functional assays and analysis for affected families to determine if the coexistence of these variants can act as genetic modifiers, which would suggest investigating their role in the damage mechanism and identifying therapeutic targets.

**Abstract:**

Marfan syndrome (MS), Loeys–Dietz syndrome (LDS), Beals–Hecht syndrome (BHS), Ehlers–Danlos syndrome (EDS), and individuals with undifferentiated connective tissue disease (UCTD) exhibit phenotypic overlap, suggesting a likelihood of genotypic coexistence. Our objective was to evaluate genetic variants (GVs), encoding 174 genes related to aortopathies, cardiomyopathies, arrhythmias, structural heart disease, and hypercholesterolemia, and their relationship to clinical and cardiovascular damage in these syndromes. This was a prospective study in Mexican patients with MS, LDS, EDS, BHS, and UCTD. One hundred and seventy-four genes related to hereditary diseases were studied using next-generation sequencing targeting coding regions. Of the 136 patients, 25 were identified with the recurrent and coexisting GV of *MYBPC3*. In the MS group, in addition to the presence of GV in *FBN1*, eight patients had GV in *MYBPC3*, six in *FBN2*, and five in *COL3A1* and *COL5A1*. In the LDS group, in addition to GV in *TGFBR1*, *TGFBR2*, and *SMAD3*, four patients presented with GV in *MYBPC3* and two with *FBN2*. In the BHS group, in addition to *FBN2*, two patients had GV in MYBPC3 and one with *TGFBR2*. In the UCTD group, nine patients had GV in *MYBPC3* and two in *COL5A1* and *COL5A2*. All syndromes coexisted with GV in genes related to arrhythmias, sarcomeres, and hypercholesterolemia. In EDS, coexistence with several sarcomere proteins was found.

## 1. Introduction

Molecular genetics has become a key approach for classifying patients with Marfan syndrome (MS) and for investigating the underlying mechanisms of the disease [1,2].

Marfan syndrome (MS) and related connective tissue disorders present heterogeneous clinical manifestations, with significant overlap in musculoskeletal, cardiovascular, and multiorgan involvement among different syndromes. This overlap makes diagnosis difficult when based solely on clinical presentation; therefore, it is important to analyze their molecular pathogenesis [3,4,5,6]. Identifying potential molecular interactions and etiological mechanisms could guide therapeutic options, which remain limited in the comprehensive management of these patients [7,8,9].

DNA-based tools such as genotyping [10] and multigene panel sequencing are currently used in research, as identifying the causative genes alone does not fully explain the diverse phenotypic spectrum of these connective tissue disorders [11].

MS is a rare autosomal dominant disease that affects both sexes, with an incidence of approximately one in 5000 to one to three in 10,000 individuals [12]. Its etiology involves genetic variants in the *FBN1* gene, which encodes fibrillin-1, an essential glycoprotein of the extracellular matrix. These variants compromise the integrity of elastic fibers [13,14,15], primarily affecting the ocular, skeletal, and cardiovascular systems.

Similarly to MS, other connective tissue disorders such as Loeys–Dietz syndrome (LDS), Beals–Hecht syndrome (BHS), Ehlers–Danlos syndrome (EDS), and undifferentiated connective tissue disease (UCTD) also present aortic dilation or dissection as the main cause of morbidity and mortality, a risk that is increased in women during pregnancy [16].

Importantly, in MS and similar conditions, in addition to aortic damage, other cardiovascular manifestations may occur concomitantly, including ventricular dysfunction [17,18], valvular disease [19,20,21,22], heart failure [23,24,25,26], and arrhythmogenic and non-arrhythmogenic cardiomyopathies [27,28], which sometimes result in unexplained sudden cardiac death [29]. These findings have prompted research into possible genetic markers associated with such outcomes [30], particularly in athletes in whom a connective tissue disorder or associated heart disease has not been previously diagnosed [31].

In MS and conditions with similar clinical manifestations, the identification of genetic variants in specific genes allows the diagnosis of most of these syndromes. However, because there is no genetically targeted treatment available to date, and the mechanisms of damage associated with the complexity of these syndromes are not fully understood, it is important to determine whether the clinical complexity could be explained by the analysis and interaction of genetic variants in genes encoding multiple proteins [32,33].

Next-generation sequencing (NGS) is an accessible, economical, and fast research technique that allows the simultaneous sequencing of hundreds of DNA regions. It generates accurate results and can be used to analyze predetermined genes that, when mutated, have been associated with an increased risk of developing a disease or a group of diseases [34,35].

Our objective was to perform an NGS study using predefined genetic panels in patients with MS and other connective tissue disorders. We included genetic variants of the genes that identify each syndrome as well as genetic variants in other genes related to aortopathies, cardiomyopathies, arrhythmias, structural cardiac abnormalities, and hypercholesterolemia. We aimed to determine whether there is a coexistence of genetic variants and to characterize the clinical and cardiovascular profiles in these patients.

## 2. Materials and Methods

### 2.1. Population Study

In a prospective observational study from 2010 to 2023, we enrolled 136 Mexican patients affected by a broad spectrum of connective tissue diseases with cardiovascular manifestations, recruited from the outpatient clinic of the National Institute of Cardiology Ignacio Chávez. All patients were evaluated by a rheumatologist specializing in this area.

**Figure 1:**
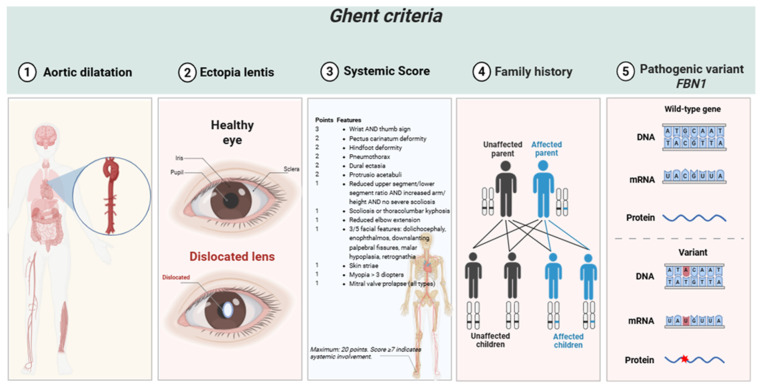
Ghent criteria: (1) aortic dilation (AD), (2) ectopia lentis (EL), (3) systemic score (SS) (>7/20 = positive for this criterion), (4) a positive family history (FH) for Marfan syndrome (MS), and (5) the determination of a positive GV in the FBN1 gene. More specific clinical data are also described for other related syndromes [37]. LDS, was identified according to the clinical criteria of each type of Loeys–Dietz, among which the following stand out: presence of hypertelorism, bifid uvula, presence of milia, arterial tortuosity, concomitant congenital heart disease, early osteoporosis, fragile skin, clubfoot, as well as being identified with the genetic variants in *TGFBR1*, *TGFBR2*, *SMAD3*, *TGFB2*, or *TGFB3* [38]. BHS, patients were identified if they presented flexion contractures in the elbows, knees, hips, ankles, and interphalangeal joints. Ear abnormalities include a flattened, wrinkled, or “snail-like” appearance, with a flattened concha and the absence of normal folds. Arachnodactyly, dolichostenomelia, and kyphoscoliosis are sometimes identified, and additional musculoskeletal features may include muscle hypoplasia, hamstring contractures, pectus excavatum/carinatum, and a high-arched palate, and are also associated with the FBN2 gene [39,40,41]. EDS was classified according to clinical phenotype whereby there are 13 types which include the classic, hypermobile, vascular xiphoscoliotic, arthrochalase, dermatosparaxis, similar to the classic, cardiac-vascular, myopathic, Musculo-contracture, periodontal, spondylocheiral dysplastic, and fragile cornea types, which are correlated with some of the following genes COL3A1, COL5A1, and COL5A2 genes [42], among others; and in subjects with undifferentiated connective tissue disease (UCTD), the analysis and description of the resulting GV are necessary since there is no known specific gene that defines them [40]. Undifferentiated connective tissue diseases (UCTDs) have been classified in this way because these patients present signs and symptoms suggestive of a connective tissue disease, but do not meet the criteria for any of the defined connective tissue diseases, for at least three years. In this case, these patients had clinical data similar to Marfan syndrome but did not meet the criteria; similarly, they did not meet the criteria for the other conditions but presented at least one clinical finding that could lead the physician to suspect MS, LDS, BHS, and EDS, among others. In the case of these patients, there is no gene that specifically identifies them. Once classified clinically according to each suspected condition, they were correlated with the type of pathogenic variant found to determine if there was a match with the gene with which each of these conditions has been associated. In individuals who did not meet the criteria for any of these diagnoses, they were considered to have UCTD, and we described the type of pathogenic variants found. In the first part of this study, we showed the usefulness of multipanel studies in diagnostic refinement [11].

Marfan syndrome (MS) was classified according to the 2010 Ghent criteria, meeting more than two of the following: (1) positive family history (FH) for MS, (2) aortic dilation (AD), (3) ectopia lentis (EL), (4) systemic score (SS) (>7/20), and (5) identification of a genetic variant in the *FBN1* gene (Figure 1) [36].

### 2.2. Sample Size

The sample size was obtained using the non-probability snowball sampling method, a sampling technique that helps researchers find samples when they are difficult to locate. This snowball sampling method works once researchers find suitable subjects who exhibit a specific symptom in patients with a rare disease. They can also recruit other affected relatives referred by the index case, thus creating a subjective sample for the study.

### 2.3. Ethical Considerations

Signed informed consent was obtained from each participant, as recommended in the Declaration of Helsinki, as amended at the Congress in Tokyo, Japan. The research was approved by the Ethics, Biosafety, and Research Committees of the National Institute of Cardiology (Project Number 23-1366). In the case of minors, verbal consent from the minor and their legal guardian was required.

### 2.4. Data Collection

Patients who had a medical examination by an expert rheumatologist and a complete medical history where relevant clinical data could be collected to classify them as having Marfan syndrome and other connective tissue disorders were included [36].

The inclusion criteria in this study were all those patients who were duly classified and diagnosed by clinical examination and the GV of the gene associated with the condition, which currently identifies them for each of the conditions and who had or did not coexist with GV of other genes that are currently known to be involved with aortopathies [43], cardiomyopathies [44,45], arrhythmias [46,47], structural alterations [48,49], and hypercholesterolemia [50].

Patients were defined as Mexican if they had at least three generations born in Mexico. Patients with aortic dilation or dissection were evaluated using magnetic resonance imaging or computed tomography. Those requiring surgery and intervention were analyzed and evaluated by a multidisciplinary cardiology team. The type of surgical technique used in each patient was included in the analysis in relation to current guidelines [51].

### 2.5. Echocardiography Study

Transthoracic echocardiography was performed using an X7-2t transducer, 2–7 mHz (Phillips, Fort Myers, FL, USA). All patients underwent a comprehensive study due to the high clinical suspicion of the variable presentation of cardiovascular alterations in these patients. Therefore, through this imaging method, the presence of other cardiovascular manifestations of MFS, such as mitral valve prolapse (MVP) and left ventricular (LV) dysfunction, which includes both systolic and diastolic function and is secondary to valvular insufficiency causing LV volume overload, as well as the presence of congestive heart failure or global cardiac dysfunction, was defined by echocardiography.

### 2.6. Computed Tomography

Our institutional protocol was performed using weight-based intravenous contrast material concentrations of 350–370 mgI/mL, administered at an injection rate of 4–5 cc/sec, followed by a saline flush of 40–50 mL. Tube current potentials were typically set at 100–120 kVp. An arterial phase was acquired using bolus-tracking automatic triggering (threshold of 120 HU) in the ascending aorta.

All data were analyzed on a dedicated workstation using Syngo Via software (v. VB40, Siemens, Erlangen, Germany). Images were loaded into a standard multiplanar cardiac reformat package and were re-constructed in the coronal, sagittal, and axial planes using a multiplanar double-oblique technique to obtain the maximal diameters of the aortic segments [52].

In patients with pectus excavatum, the Haller index was determined by measurements calculated by magnetic resonance imaging, which was calculated by dividing the maximum transverse diameter of the thorax by the minimum anteroposterior diameter, which goes from the anterior edge of the vertebral body to the posterior edge of the sternum [53].

### 2.7. Sample Collection and Genomic DNA Extraction

A quantity of 20 mL of blood was obtained upon admission and 48 h after treatment. Samples were identified as pre (0 h) or post sample (48 h). They were centrifuged at 3000 rpm for 20 min at 4 °C. Serum was stored in three or four Eppendorf aliquots of 1.5 mL and stored at <70° until processed. Samples were also taken for analysis of a complete blood count and blood chemistry. Peripheral blood samples were obtained by venipuncture in tubes with sodium ethylenediaminetetraacetic acid (Na-EDTA). DNA extraction was performed using the saline expulsion technique. Leukocytes were separated from whole blood by lysis of erythrocytes with 1X lysis solution (SLR). Subsequently, the leukocyte pack was incubated with 10% SDS and proteinase K (10 mg/mL) at 37 °C overnight for enzymatic digestion. Finally, the DNA was quantified in a spectrophotometer (Bio Photometer Plus, Eppendorf Hamburg, Germany) at a wavelength of 260/280 nm.

### 2.8. Next-Generation Sequencing (NGS)

DNA samples were sequenced using the TruSight™ Cardio Sequencing Panel from Illumina, (San Diego CA, USA) which provides approximately 99% coverage, achieving an average coverage depth greater than 200× of coding regions across 174 genes associated with cardiovascular diseases, including cardiomyopathies, arrhythmias, aortopathies, structural heart defects, and familial hypercholesterolemia. Library preparation was carried out following the manufacturer’s instructions. DNA fragment size was assessed using the High Sensitivity 474 microchip kit (HS-NGS), (Agilent, Technologies, Santa Clara, CA, USA) confirming an average fragment size appropriate for sequencing. Libraries were normalized to a final concentration of 4 nM and loaded into the sequencing cartridge.

Sequencing was performed on the Illumina NextSeq 550 platform using paired-end reads of 150 bp. Quality control and preliminary data analysis were conducted on the Illumina BaseSpace server (https://basespace.illumina.com/home, accessed on: 15 February 2022). Sequencing quality was assessed by Phred score and reads with scores below 30 were excluded from downstream analysis. All sequencing runs included negative controls and PhiX internal controls to monitor background signal, sequencing error rates, and potential cross-contamination. No evidence of contamination or barcode bleeding was detected.

### 2.9. Variant Analysis and Annotation

Alignment of sequencing reads to the GRCh38 human reference genome was performed, and the resulting BAM files were archived in the NCBI repository (https://www.ncbi.nlm.nih.gov/bioproject/PRJNA931345, accessed on 3 February 2023). We performed variant calling with DRAGEN Enrichment Software, v3.4 (Illumina, San Diego, CA, USA) a powerful tool for detecting genetic variants with high accuracy. This software uses stringent filtering criteria to retain only variants that pass rigorous quality thresholds, including read depth, base quality, mapping quality, and strand bias. The Variant Interpreter platform was used (https://support.illumina.com/sequencing/sequencing_software/basespace-variant-interpreter.html) accessed on 7 March 2023.

Both rare variants (with a frequency lower than 1%) and variants with a frequency greater than 1% in population databases such as TOPMed, the 1000 Genomes Project, and the NHLBI Exome Sequencing Project were retained. Utilizing the ClinVar database (https://www.ncbi.nlm.nih.gov/clinvar/, accessed on 18 August 2023), which aggregates genomic variation and its relation to human health, we classified the identified genetic variants as pathogenic, likely pathogenic, or variants of uncertain significance (VUS). These classifications follow the Standards and Guidelines for the Interpretation of Sequence Variants as recommended by the consensus of the American College of Medical Genetics and Genomics and the Association for Molecular Pathology [54]. All genetic variants were reported following the Human Genome Variation Society (HGVS) nomenclature for standardized variant description [55].

A comprehensive Supplementary File S1 containing all reported variants, including rsIDs, HGVS nomenclature, exon localization, mutation type, and clinical classification (pathogenic, likely pathogenic, or VUS), was generated for all patients included in the study (Supplementary File S1).

### 2.10. Protein–Protein Interaction (PPI) Network Construction

To explore functional relationships among proteins encoded by the genes of interest, a protein–protein interaction (PPI) network was constructed using the STRING database (version 12.0). Network construction incorporated the genes of interest and applied a high-confidence interaction threshold (score ≥ 0.7). Multiple evidence streams were integrated, including experimentally validated interactions from curated databases, manually annotated pathways and complexes, gene co-expression patterns, and automated text mining of the scientific literature. The resulting network contained 62 nodes and 1230 edges, with an average node degree of 39.7 and an average local clustering coefficient of 0.855. The network had significantly more interactions than expected by chance (PPI enrichment *p*-value < 1.0 × 10^−16^). This analysis allowed for the investigation of potential biological pathways and interaction clusters relevant to cardiovascular pathophysiology.

## 3. Results

### 3.1. Study Population

One hundred and thirty-six patients were included 80 women (58.8%) and 56 men (41.2%), with a median age of 24 years (Q1–Q3) [18,19,20,21,22,23,24,25,26,27,28,29,30,31,32,33,34]. Table 1 shows the demographic characteristics, comorbidity frequencies, and Ghent criteria for each of the connective tissue disorders studied.

#### Coexistence of Genetic Variants with Different Genes

Table 2 shows the frequencies of the coexistence of variants of these genes in relation to those already associated with each of the syndromes.

- Detailed information regarding each identified genetic variant, including transcript annotation, exon localization, mutation type, and pathogenicity classification according to ClinVar and HGVS nomenclature, is provided in the Supplementary File S1.
- Marfan syndrome: A total of 64 patients with MS, 39 (61%) women and 25 (39%) men, with a median age 26 (Q1–Q3) (20–35), had a prevalence of *FBN1* in 62 (96.8%), of whom eight patients (12.5%) did not have coexisting abnormalities with other genes, and in 56 (87.5%), the *FBN1* gene coexisted with variants of other genes.
- Loeys–Dietz syndrome: In the 16 patients with LDS, nine (56%) were women and seven (44%) men, with a median age of 23 (Q1–Q2) (21–41), of whom 13 (81.2%) had prevalence of *TGFBR2* and three (18.8%) of *TGFBR1*. Of the total cases, one (6.2%) did not coexist with another gene, and in 15 (93.7%), coexistence with GV of other genes was found.
- Beals–Hecht syndrome: In 13 patients with BSH, five (38.5%) were female and eight (61.5%) male, with a median age of 27 years (Q1–Q3) (13–32). All 13 (100%) had a positive GV in *FBN2*, and all had coexisting GV.
- Ehlers–Danlos syndrome (EDS): In seven patients with Ehlers–Danlos, four (57%) were female and three (43%) male, with a median age of 20 years (Q1–Q3) (15–28). Of these, four (57.1%) had GV in the *COL3A1* gene, two (28.5%) in COL5A2, and one (14.2%) in COL5A1, and all had coexisting GV with other genes.
- Undifferentiated connective tissue disease: In the 35 patients classified with UCTD, 22 (62.8%) were women and 13 (37.2%) were men; the median age was 21 years (Q1-Q3) (17–31). The frequencies of GV were nine (25.7%) in *MYBPC3*, seven (20%) in *SDHA*, five (14.2%) in *AKAP9*, five (14.2%) in *RYR2*, four (11.4%) in *TTN*, three (8.5%) in *DES*, three (8.5%) in *NOTCH1*, two (5.7%) in each of the following: *TNNT2*, *MYPN1*, *PRDM16*, *RYR1*, and *ZBTB17*, and one (2.8%) in each of the following: *DSP*, *TTN-ASI*, *COL5A1*, *COL5A2*, *HFE*, *SOS1*, *HCN4*, *LAMA2*, *SGCB*, *SMAD3*, *TRDM*, *GJA5*, *TPM1*, *HADHA*, *ZHX3*, *ACTN2*, *KCNH2*, and *ACTA1.*

### 3.2. Coexistence of Genetic Variants MYBPC3

The most frequent coexisting condition across all disorders was with the *MYBPC3* variant. In MS, the frequency of variants in the *MYBPC3* gene was found in eight (12.5%), in LDS in six (37.5%), in BHS in two (15.3%), and in UCTD in seven (20%). All individuals shared a recurrent variant of the NM_000256.3:c.2440_2442del, p.Lys814del Inframe deletion. Patients with EDS did not have coexisting variants with this gene. The complete list of recurrent and coexisting variants identified across all connective tissue disorders is summarized in the Supplementary File S1. This dataset includes standardized HGVS annotation and variant pathogenicity classification.

#### 3.2.1. Coexistence of Genetic Variants MYBPC3 in Marfan

Table 3 describes the type of genetic variants coexisting with the *FBN1* gene and the type of cardiovascular disease in patients with MS. In eight patients with coexisting GV associated with the *MYBPC3* gene, all had aortic dissection (AoD), and two of them had aortic dissection, one occurring in a woman in the postpartum period and the other in a man with a 44 mm aortic dilation (AD) and severe left ventricular dysfunction with an ejection fraction (LVEF) of 26%. Four patients had mitral regurgitation, one of whom was associated with mitral valve prolapse (MVP), one had eccentric left ventricular hypertrophy, and another had concentric left ventricular hypertrophy. In four patients with coexisting GV associated with *FBN1* and transient tachycardia (*TTN*), four had AD and one had arrhythmogenic cardiomyopathy.

In eight subjects with coexisting variant genetics of collagen genes, one with variant of *FBN1*, *COL5A1*, and *JUP* had septal hypertrophic cardiomyopathy without outflow tract obstruction, and another patient had descending aortic and abdominal aneurysms; both also had pulmonary vascular shunts (PVSs). In six subjects with *FBN1* and PVS of *FBN2*, three had aortic dissection (AD); in one woman, the pregnancy had to be terminated at 37 weeks. In two subjects with GV of *FBN1* and also the *TGFBR2* gene, one was female, and the pregnancy had to be terminated at 34.3 weeks due to aortic dilation of 60 mm; in the other case, cardiovascular data were unavailable because the patient did not continue with comprehensive evaluation. Other cardiovascular data associated with other genes are also shown. The Supplementary File S1 provides the complete curated variant dataset used in this study, including gene annotation, HGVS nomenclature, exon information, mutation category, and clinical interpretation.

#### 3.2.2. Coexistence of Genetic Variant MYBPC3 in Loeys–Dietz, Beal–Shecht and Ehlers–Danlos Syndromes

This study also describes the cardiovascular findings in the LDS group, where 7/16 (43.7%) presented with coexisting *MYBPC3*. Supplementary Table S1. In three patients, their cardiovascular evaluation was unknown because they did not attend the comprehensive evaluation and follow-up. One patient with a pulmonary vascular lesion associated with *TGFBR2+MYBPC3+MYP06* had a bicuspid aortic valve and an anomalous origin of the left subclavian artery. Two patients with *TGFBR2+MYBPC+NOTCH1* presented with a ventricular septal defect (VSD) in one and an anomalous origin of the left subclavian artery in the other. One patient with *TGFBR2+MYBPC3+HFE* presented with pulmonary artery dilation and dissection with extensive late gadolinium enhancement at the left and right ventricular junction. The coexistence of other genes in this group of patients is also shown. One patient with a GV associated with *TGFBR2* and no coexisting GV had congenital heart disease (CHD) and underwent coarctectomy.

Of the 13 cases with BHS, two (15.3%) had coexisting *MYBPC3* GV; one showed mild aortic dissection, eccentric left ventricular hypertrophy, and mitral valve prolapse, while the cardiovascular evaluation of the other was unknown. In 4/13 (30.7%), in addition to the FBN2 gene, there was the coexistence with other GV genes of sarcomere genes such as TTN, TTN-ASI, DSP, and PDLIM3 of SCN2B sodium channels; mitochondrial enzyme genes such as SDHA; genes with function in apolipoproteins such as *APOC2* and *APOC4*; and Golgi apparatus genes such as GV of FKRP. In patients with Ehlers–Danlos syndrome, no GV was found in *MYBPC3*; however, one subject had a GV in *COL3A1*+TTN, one with *COL3A1* and a GV of *MYLK* (a gene associated with myosin chains), one with COL5A2 and a GV of mitochondrial genes such as SDHA and potassium channels such as HCN4A, and one with the coexistence of *COL5A1* and a GV of genes involved in signaling processes such as *JAG1*. In mitochondrial processes such as DNAJC19, a GV of laminin such as *LAMA2* is present.

#### 3.2.3. Coexistence of Genetic Variant MYBPC3 in Undifferentiated Connective Tissue Disease

In Supplementary Table S2, the subjects with undifferentiated connective tissue disease were shown to be non-positive for *FBN1*, *TGFBR1*, *TGFBR2*, *SMAD3*, and *FBN2*. They presented only one Ghent criterion in 24/35 (68.5%), of whom 14 (58.3%) had positive SS, three (8.3%) had DAo, and seven (50%) had a family history of a condition classified as MS. Two individuals presented with two criteria: DAo and SS. One patient had coexisting GV of *AKAP9* and *COL5A2* without relevant cardiovascular damage on imaging but presented with dysautonomia. Another subject with *RYR2* and *COL5A1* GV did not receive cardiovascular evaluation. In this group of patients, nine (25.7%) had sarcomere gene variants (SGVs) such as *MYBPC3*; two had no other coexisting variants, two also had a mitochondrial gene variant such as *SDHA*, two had other GV of sarcomere genes *DES* and *DSP*, and one had a variant of triadin-encoding genes such as *TRDN*. There were also five (14.2%) subjects with *TTN* SGVs; one coexisted with *SDHA*, and one with *ZBTB17*. The heterogeneous coexistence of SGVs is shown, and SGVs of the gene encoding *AKAP9* (A-Kinase Anchoring Protein 9) and the gene encoding *RYR2* (Ryanodine Receptor 2) were also found. The comprehensive curated Supplementary File S1 generated in this study may facilitate future genotype–phenotype correlation analyses and functional studies aimed at understanding the role of coexisting variants as potential genetic modifiers in connective tissue disorders with cardiovascular involvement.

#### 3.2.4. Implications of Cardiovascular Damage in Marfan Syndrome and Other Connective Tissue Disorders

Table 4 shows the frequency of cardiovascular damage found in MS and each of the syndromes and subjects with undifferentiated connective tissue disease.

Of the 136 (12.5%) subjects with *MYBPC3*+ who had undergone complete imaging studies, 17 were matched by age, sex, and condition to comparatively analyze left ventricular ejection fraction and aortic diameters. The presence of dissection, deaths, and types of surgery required were also analyzed through a sub-analysis in each group, revealing the following findings in Table 5.

Table 5 shows that a sub-analysis was performed comparing *MYBPC3*-positive patients versus age- and sex-matched subjects who did not have *MYBPC3*.

In the analysis of this group, 3/17 (17.6%) who were *MYBPC3*-positive presented with dissection, two died 2/17 (11.7%), and in those who were *MYBPC3* negative, none had dissection and all survived. Of the three patients who presented with dissection, two had MS and one had LDS. Table 6 shows the type of surgery performed on subjects positive for *MYBPC3* in coexistence with the gene that identifies each condition and subjects negative for *MYBPC3*, who only presented the gene that identifies each condition.

### 3.3. Sub-Analysis of Genetic Coexistence vs. Non-Coexistence, Regardless of the Type of Coexisting Gene

In the group of patients with MFS, we found that patients with genetic coexistence (n = 53) had a lower left ventricular ejection fraction (LVEF) with a median (Min–Max) of 55 (24–65) versus cases without coexistence (n = 7) and a median of 58 (50–59). This difference was not statistically significant (*p* = 0.28). When both groups (with and without genetic coexistence) were compared, the number of subjects with preserved LVEF was 43 (81%) vs. seven (100%) (*p* = 0.001). Furthermore, in those with coexistence, seven (13%) subjects had moderately reduced LVEF and three (5.6%) had reduced LVEF, compared to no patients in this condition in those without genetic coexistence.

The same pattern was observed in LDS, where the number of cases with genetic coexistence was eight vs. two without. However, we showed that subjects with coexistence tended to have a lower LVEF, with a median of 50 (34–60) vs. 54 in cases without coexistence. This difference cannot be statistically analyzed. Similarly, among the subjects with genetic coexistence, three (37.5%) had a moderately reduced LVEF and one (12.5%) had a reduced LVEF.

In subjects with EDS and BHS, all cases had genetic coexistence; there were no cases without coexistence. However, in both groups, LVEF was preserved in all cases.

And in the UCTD cases, where there were 11 with coexistence and seven without genetic coexistence; no differences were found, except that one individual in the genetic coexistence group had a moderately reduced LVEF.

### 3.4. We Show Cases of Subjects with Clinical Relevance

**Figure 2:**
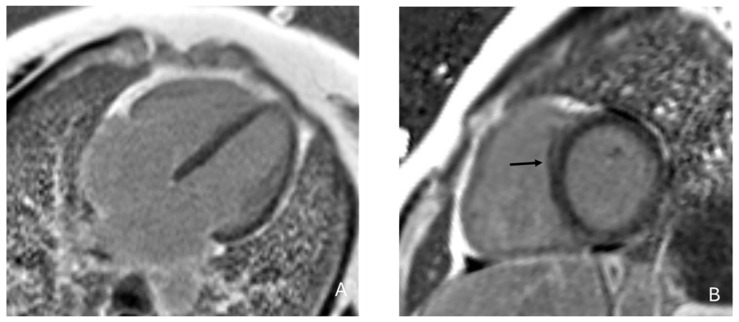
MRI inversion-recovery sequence in four chambers (**A**) and basal short axis (**B**). The black arrow shows late enhancement mid-wall at the septum.

Case 1 involves a 54-year-old woman with coexisting pulmonary venous thrombosis (PVT) in *FBN1*, *MYPN*, and *MYBPC3*. Her family history includes a mother with Marfan syndrome (MFS), and at age 3, she suffered a retinal detachment, leading the ophthal-mologist to suspect MFS. She presents with subluxation of both lenses, overlapping teeth, a high-arched palate, retrognathia, arachnodactyly, pectus carinatum, and dural ectasia. During her clinical course, she has been asymptomatic, and her current echocardiogram shows a normal left ventricular ejection fraction (LVEF) of 63% and a strain global longitudinal (SGL) of 19.9. However, cardiac magnetic resonance imaging revealed abdominal aortic dilation and diffuse fibrosis, as well as pulmonary trunk dilation (Video S1, Figure 2).

**Figure 3:**
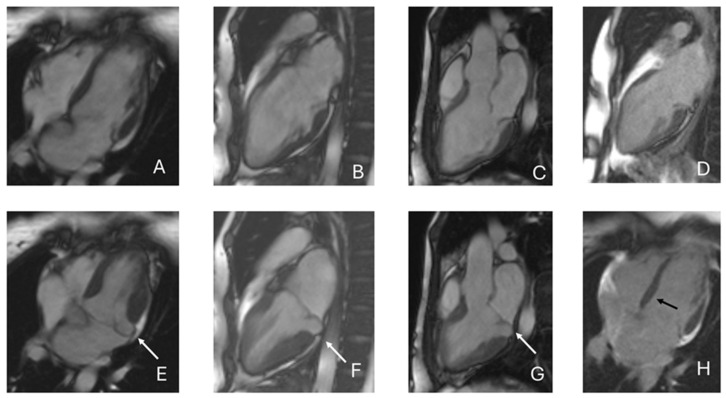
Cardiac magnetic resonance imaging. Cine in 4 (**A**), 2 (**B**), and 3 chambers (**C**) in diastole (upper row) and systole (bottom row). The aortic root has a diameter of 41 mm at the Valsalva sinus. The right ventricle with an ejection fraction of 43%. In (**D**–**H**) inversion- recovery sequence. In (**E**–**G**) the white arrow shows a submitral aneurysm, In (**E**) with subtle mid wall late and in (**H**) enhancement in the septum (black arrow).

Case 2 is a 33-year-old woman with no family history of MS. We found a positive ventricular tachycardia (PVT) on *FBN1+TTN*. Her medical history includes a mother with a history of unexamined tachycardia’s and a brother who died in infancy two days after birth and presented with tachycardia. At age 6, a decrease in visual acuity was noticed, and an ophthalmological evaluation revealed lens dislocation, leading to a diagnosis of MS. She never had clinical follow-up. At age 20, she presented to our center for the first time with palpitations and severe back pain. A low-frequency ventricular arrhythmia with a single ectopic focus was documented, presenting as an isolated pattern with regular ventricular coupling intervals. The Holter monitor showed monomorphic extra heart sounds in less than 1% of cases and one triplet episode. Magnetic resonance imaging showed aortic root dilation, subvalvular mitral aneurysm, left atrial dilation, keel chest, left ventricular systolic function (LVEF) 46, and posterior leaflet prolapse with Barlow’s disease. At age 24, he underwent mitral valve replacement with a St. Jude Masters 31 mechanical prosthesis and aortic valve re-encapsulation surgery (Florida sleeve technique). Six years later, at age 30, he developed left hemiparesis, and an intracranial aneurysm was found. He remained under neurological surveillance. At age 32, he presented with acute coronary syndrome attributed to an embolism in the left anterior descending artery (LAD), which was related to his adherence to anticoagulants (Video S2, Figure 3).

**Figure 4:**
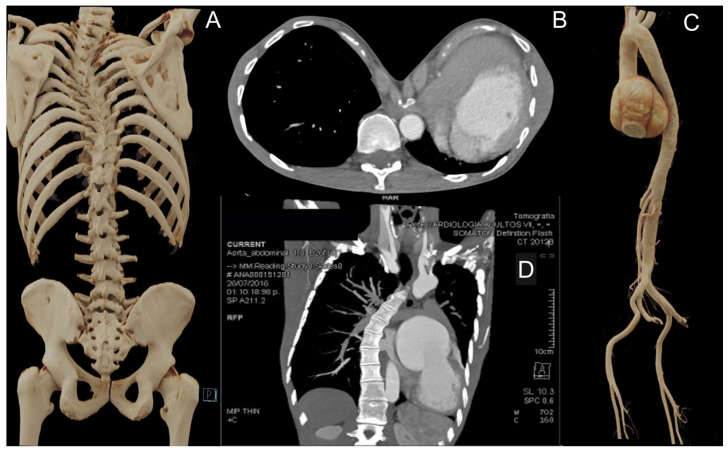
Computed tomography angiography (CTA) of the chest. (**A**) 3D cinematic volume rendering reconstruction (VRT) of the thoracic skeleton (posterior view) and (**B**) axial view demonstrating thoracic rotoscoliosis and thoracic cage deformity. (**C**) Cinematic volume rendering reconstruction (VRT) of the thoracoabdominal aorta, demonstrating severe dilation of the aortic root. We showed how the heart was deviated to the left, and this conditioned restriction of the volume of the left lung; it had left bronchial compression due to entrapment by aortic dilation and a maximum of 84 mm in the sinuses of Valsalva (**D**).

Case 3: A 44-year-old man who noticed decreased visual acuity consulted an ophthalmologist who found lens dislocation and an aortic murmur, which is why he was referred to our institute. He had no diagnosis of MS. The patient reported no family history of MS; however, his mother died suddenly at age 40. On examination, he was 1.88 m tall with a 1.93 m arm span. Facial features included a positive Walker–Murdock sign, scoliosis, striae, and pectus excavatum. The computed tomography scan revealed a Haller index of 53, which causes displacement of the heart to the left and restriction of left lung volume, left bronchial compression due to entrapment, aortic dilation, and a maximum of 84 mm in the sinuses of Valsalva. The genomic study showed the coexistence of GV of the *FBN1* and *SCN5A* genes (Figure 4).

**Figure 5:**
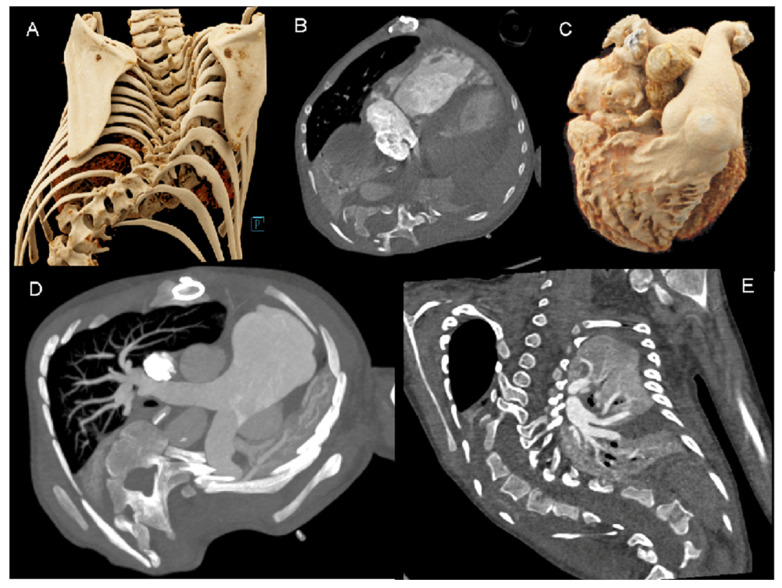
Computed tomography angiography (CTA) of the chest. (**A**) 3D cinematic volume rendering reconstruction (VRT) of the thoracic skeleton (posterior view) demonstrating severe thoracic rotoscoliosis and thoracic cage deformity. (**B**) Axial view demonstrating pectus carinatum. (**C**) 3D cinematic volume rendering reconstruction (VRT) of the heart (anterior view) and (**D**) axial view demonstrating severe dilation of the pulmonary artery. (**E**) Coronal view demonstrating severe rotoscoliosis and atelectasis of the left lung with mild pleural effusion.

Case 4 presents the case of a child admitted to the Institute at 3 years and 8 months of age. During the first year of life, MS was suspected, but no genomic study was performed. At age 3, the child began experiencing dyspnea and was seen at a pediatric hospital, where a heart murmur was detected. The child was then referred to our institute for evaluation. Based on the Ghent criteria, the child met the following: (1) Systemic score of 11/20 points: Facial features, Steinberg–Walker–Murdock syndrome, rib cage deformity, severe scoliosis, mitral valve prolapse, dural ectasia, flat feet, hypermobility, and hyperlaxity. (2) The CT scan revealed aortic dilation at the level of the sinuses of Valsalva, mitral valve prolapses, moderate mitral and tricuspid regurgitation, and dextro-converted scoliosis. (3) Lens subluxation in both eyes. Fourth criterion: negative; there was no family history of MS, and the genetic study was negative for the *FBN1* GV gene and the *COL5A2*, *SDHA*, and *HCN4A* genes. In conclusion, he met three Ghent criteria but was not positive for the gene associated with MS. The echocardiogram reported an LVEF of 59% and mitral and tricuspid valve prolapse. At age 4, he underwent David surgery, mitral and tricuspid valve repair. At age 8, he underwent aortic valve replacement due to severe aortic insufficiency. At 13 years of age, he presented with aortic and mitral prosthetic valve dysfunction, for which aortic valve replacement was performed, followed by the subsequent enlargement of the aortic annulus (Manougian type), removal of the mitral annulus plus mitral valve replacement with Carbiomedics prosthesis, and the reconstruction of the interatrial septum with atrial fenestration. The patient’s diagnosis is Ehlers–Danlos syndrome (Figure 5).

### 3.5. Construction of a Protein–Protein Interaction (PPI) Network In Silico

**Figure 6:**
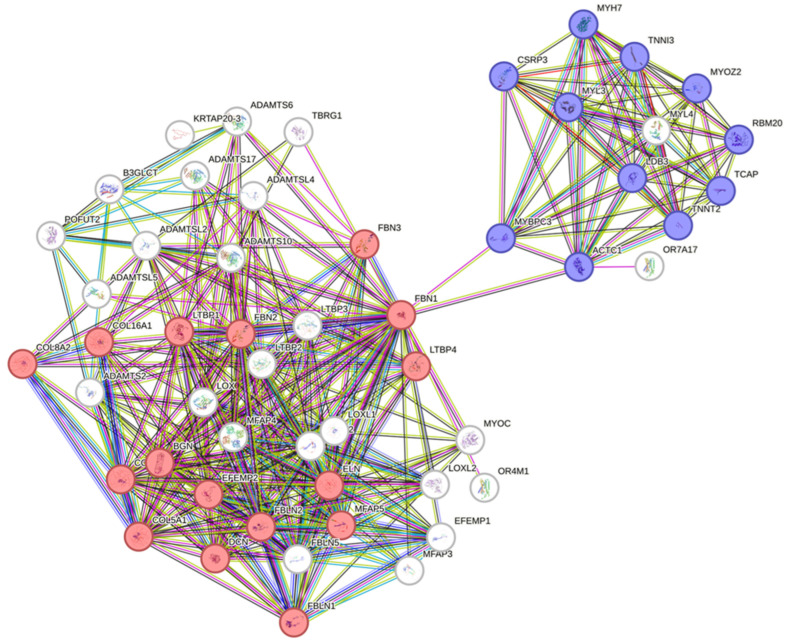
Protein–protein interaction network (PPI) with a PPI enrichment *p*-value of <1.0 × 10^−16^, generated using the STRING database. The nodes represent proteins, and the edges indicate known physical or functional associations. The proteins associated with hypertrophic cardiomyopathy are highlighted in purple, while those involved in connective tissue disorders are marked in red.

**Figure 7:**
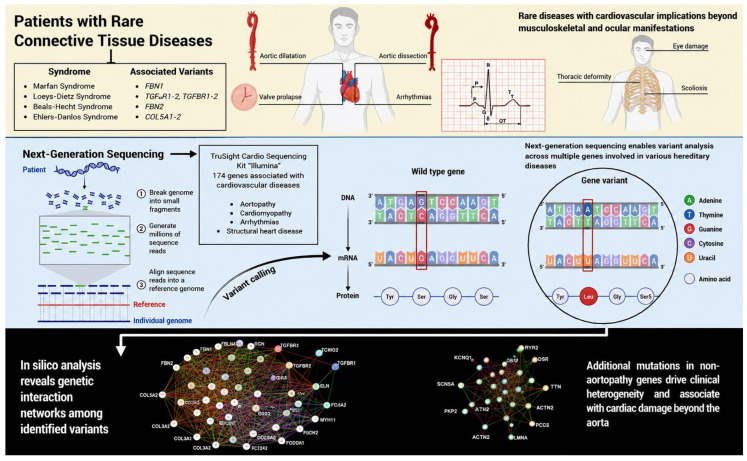
Visual summary shows that in rare diseases, delayed diagnosis leads to a poor prognosis. There is a gap in knowledge regarding the various genetic interactions that may explain the overlap and clinical variability in these syndromes. Next-generation sequencing (NGS) allows us to approach the investigation of genetic coexistence, which should be validated by studying the function of these interactions and coexistences in the genetics variants. This could provide valuable information on mechanisms of damage and therapeutic targets.

Figure 6 shows a protein–protein interaction (PPI) network generated using the STRING database, with a PPI enrichment *p*-value < 1.0 × 10^−16^. Each node represents a protein, and the borders indicate known functional or physical interactions. Two distinct groups are observed based on node color: red nodes correspond to proteins involved in connective tissue disorders, and purple nodes represent proteins associated with hypertrophic cardiomyopathy. The FBN1 protein is centrally located within the red group and shows a direct interaction with *MYBPC3*, located in the purple group. This link connects the two functional modules and suggests a possible functional interface between extracellular structural integrity and sarcomeric function. In Figure 7 we present a visual summary of the study.

## 4. Discussion

Rare syndromes of genetic etiology, phenotypic and genotypic heterogeneity, with systemic and cardiovascular damage, can present in mild, complex, or catastrophic forms, and this variability has been observed even within the same family [56].

Patients with MS and similar conditions who present with a combination of cardiovascular damage, such as aortic dilation and/or dissection, as well as valvular disease, ventricular dysfunction, heart failure, and cardiopulmonary problems due to distortion of the cardiac area or thoracic compression and deformity, and cardiac rhythm disorders, experience a complicated evolution and an uncertain prognosis [57,58,59].

The complex findings of cardiovascular and musculoskeletal damage in this study confirm what has been reported in isolated cases, suggesting that clinical phenotypic heterogeneity may be linked not only to the genes primarily associated with each syndrome but also to other multifactorial mechanisms, within which genetic or protein interactions as underlying mechanisms require further attention in research [32].

The presence in the same subject of GV of various genes in addition to the GV of the gene that identifies the connective tissue syndrome they suffer from could justify the clinical overlap in the same subject and between MS and the different syndromes, which confuses the clinician when the classification of these patients is based only on criteria obtained by inspection, examination, and imaging [60].

Attempting to arrive at a diagnosis through nosology is undoubtedly very helpful but limited; however, adding expanded genetic testing could lead not only to a precise diagnosis but also to a better explanation of the complexity a patient may present and, in research, to clarify the possible sites of involvement in the damage mechanism or for therapeutic intervention [61,62,63].

In the mechanisms of MS, fibrillin-1 plays a key role in the regulation and availability of TGF-β [64,65,66]. Several variants of this gene also lead to loss of extracellular matrix (ECM) homeostasis. The loss of fibrillin-1’s ability to remain inactive by binding to TGF-β [66] and the latent binding protein increases TGF-β ligand levels in the extracellular space, which alters its functionality, as it is a molecule that regulates several processes, including apoptosis, collagen production, and participation in extracellular matrix (ECM) remodeling [67,68].

The loss of regulation of TGF-β has been shown to interfere with the maintenance and homeostasis of the aortic wall, and this effect is not only subject to the bioavailability of TGF-β in aortic tissue. It is possible that other factors play an additional role in MS that have not been comprehensively studied. The analysis and characterization of these potential factors could be a line of research to pursue in order to explain the differences in the phenotype of aortic damage in patients with MS.

The identification in this study of new genes that could modulate or modify active TGF-β levels could lead us to a better understanding of the variability in the cardiovascular phenotype of patients with MS and other connective tissue disorders.

Next-generation sequencing analyses allow for the inclusion of intronic regions already known to be involved in the damage of certain hereditary diseases. It may be necessary to complement the multipanel study by including not only those genes already known to be involved in cardiovascular damage but also those implicated in musculoskeletal and ocular damage. This could further enhance our understanding of the diversity in the expression of syndromic disease.

Therefore, the results of this study on the coexistence of GV in various genes in the same patient suggest that this interaction could also explain, in addition to possible underlying mechanisms of damage, the significant clinical heterogeneity resulting from the combination of alterations in various organs and systems. It remains to be explained whether this complexity also influences the evolution, type of outcomes, and prognosis of these conditions.

It should be noted, however, that the TruSight Cardio panel used in this study is specifically designed to target genes associated with cardiomyopathies and arrhythmias. Therefore, in this series, the *MYBPC3* gene variant was frequently found in subjects with MS and cardiomyopathy. This is interesting because these variants have been associated with hypertrophic cardiomyopathy, a condition in which nonsense mutations in cMyBP-C occur. This has led to the suggestion of evaluating its effect on the interaction with thin filaments and myosin and on protein stability to explain how they could lead to hypertrophic cardiomyopathy or other types of clinical presentations. Many subjects with this variant in this gene have outcomes ranging from being asymptomatic to the presence of hypertrophic cardiomyopathy, fibrosis, rhythm disorders, and sudden death. We also found in some patients the coexistence of several GV that encode for other sarcomere proteins such as *TTN* and *DES*, a finding that leads us to explore whether the alterations in cardiac rhythm observed in these patients and, in addition, those not classified as UCTD converge with GV involved in the sarcomere structure, since in this latter undifferentiated group the frequency of these GV was high.

The frequency of GV found in *MYBPC3*, which encodes cardiac myosin-binding protein C (cMyBP-C), a critical component of the sarcomere, could be considered a phenotype modifier, as has already been described in the *FBN1* gene [56].

Experimental studies of murine null models for *MYBPC3* have found that they exhibit left ventricular dilation, myocardial fibrosis, and impaired contractility [69].

In this study, we did not find evidence of hypertrophic cardiomyopathy, although fibrosis was observed in four (6.2%) subjects with MS and one (7.6%) with SBH, which could be a subclinical finding.

One patient with SBH was shown to have arrhythmogenic cardiomyopathy, and the genetic result was a positive GV for *FBN1* and a GV for *TTN* rs770301886 (VUS).

Our in silico analysis of the PPI network revealed possible functional links between sarcomeric proteins such as *MYBPC3* and extracellular matrix (ECM) components such as FBN1. This connection generates the hypothesis of a functional interface between the extracellular structure and cardiomyocyte contractility, but this requires experimental validation [70].

Recent studies suggest that, in addition to extracellular matrix (ECM)-related proteins such as FBN1, several proteins influence cytoskeleton architecture and intracellular signaling [71]. These interactions, especially in focal adhesions and caveolae, link membrane receptors to actin filaments and play a key role in mechanotransduction [72,73,74,75].

This complexity may contribute to variable cardiovascular phenotypes in connective tissue disorders. Evidence from biochemical and genetic studies shows that cytoskeletal components interact with elements of signaling pathways, including ribosomes and mRNA, suggesting a regulatory role in protein translation and localization [76,77]. These processes can influence cell structure and function, especially under pathological stress [78,79].

On the other hand, mitochondrial dynamics are closely linked to the cytoskeleton, and mitochondrial reorganization affects mitochondrial morphology, positioning, and energy production, which together provide a plausible link between extracellular matrix (ECM) remodeling, cytoskeletal disruption, and myocardial vulnerability.

The interaction between *MYBPC3* and *FBN1* observed in the in silico network supports the hypothesis that alterations in ECM integrity can sensitize cardiomyocytes to intracellular contractile dysfunction [80].

The network architecture we analyzed provides GV in sarcomere genes that could be involved in the pathophysiology of cardiomyopathies associated with connective tissue disorders. However, as this is a computational prediction based on aggregated data, it does not demonstrate that these interactions are physiologically operative in our patients. The presence of dual-impact GV may constitute a possible mechanistic basis and highlights the need for comprehensive genetic screening in at-risk populations, but functional validation is required.

The *FBN1* gene in MS is frequently associated with dilated or dissected aortic disease; however, this condition also occurs in other syndromes such as Loeys–Dietz syndrome, which presents mutations in other genes such as *TGFBR1*, *TGFBR2*, *SMAD3*, *TGFB2*, *TGFB3*, and vascular type EDS, and also in non-syndromic conditions related to GV in other genes such as *ACTA2*, *MYH11*, *MYLK*, *LOX*, and *PRKG1*. The relevant point is that in some cases, such as in LDS, aortic dilation is progressive, and dissection occurs even in diameters smaller than 40 mm. On the other hand, aneurysms may be present in other sites outside the aorta, which is more frequent in LDS and EDS than in other connective tissue disorders [81,82].

Detailed screening of the proband leads to different results in these patients, and GV interactions in one or more genes have already been reported, so double mutations in different genes can result in more severe phenotypes; therefore, evaluating copy number variation could optimize the diagnosis of hereditary diseases [83].

The interaction of GV between genes in the context of myocardial fibrosis is a key feature of cardiac remodeling and contributes to diastolic dysfunction and arrhythmias. In myocardial fibrosis, there is proliferation of cardiac fibroblasts and excessive collagen deposition [84]. Fibrosis can be mediated by fibroblast activation via the TGF-β1/Smad2/3 pathway [85]. It is important to note that *TGFBR2*, a receptor for *TGFβ1*, is frequently mutated in syndromes such as Loeys–Dietz, which may suggest a pathogenic link when it co-participates with the GV of sarcomeres’ genes such as *MYBPC3* [86]. In this study, we found asymptomatic patients with subclinical myocardial fibrosis, which suggests a comprehensive study in them if we consider these coexisting conditions.

Sudden death occurring during sports activity is rare; however, its association with connective tissue disorders and structural and electrical cardiac abnormalities is well-documented [87].

Common causes related to this condition include coronary artery disease, muscular bridging, congenital coronary artery anomalies, subarachnoid hemorrhage, arrhythmogenic right ventricular dysplasia, aortic coarctation, myocarditis, pulmonary embolism, aortic stenosis, mitral valve prolapse, and Wolff–Parkinson–White (WPW) syndrome. The estimated risk of sudden death during sports ranges from one to three deaths per 100,000 people per year [87,88,89].

The conditions most often overlooked as causes of sudden death are hypertrophic cardiomyopathy, connective tissue disorders, and coronary anomalies. Therefore, although widespread screening is not considered cost-effective given the low incidence (1.82/1000), genetic testing in individuals with hereditary syndromes or a family history of sudden cardiac death is strongly justified [90].

The coexistence of MS and HCM had already been described in 1988 in one case. It was proposed that in subjects with MS the loose structure of the connective tissue of the mitral valve, which causes mitral valve prolapse, could also contribute to myocardial disease, since disorganized collagen fibrils and the accumulation of acidic mucopolysaccharides have been found in the myocardial stroma, suggesting subclinical myocardial involvement [91].

In this series, we also had patients without *MYBPC3* GV; however, some had GV of multiple genes. The possibility of a synergistic effect between the GV of genes such as *FBN1*, *TGFBR1*, *TGFBR2*, *FBN2*, and *SMAD* with other mutations requires confirmation, as our finding was interesting. In one subject with GV in the *FBN1* gene plus *MYBPC3* plus MYPN1 (the latter encoding a protein that interacts with nebulin in skeletal muscle or nebulet in cardiac muscle), the skeletal muscle damage was severe, and the subject also had lens dislocation, aortic dilation, mitral valve prolapse, and pulmonary artery dilation.

Another coexisting GV in these patients was *FBN1*+*RBM20*. The latter is a gene that codes for the RBM20 protein, which is crucial for the regulation of the alternative splicing of titin, a giant protein of the cardiac muscle. If a mutation occurs in the *RBM20* gene, its function is altered, leading to progressive cardiac dysfunction, enlargement of the heart, and finally heart failure. Diffuse fibrosis was found by magnetic resonance imaging; we consider that it could be a subclinical state where evaluating genetic risk could be a priority.

The patient found to have a positive *FBN1* mutation GV for the *SCN5A* gene was diagnosed with multiple myeloma (MM) upon admission at age 44, when he began experiencing dyspnea. This patient presented with severe musculoskeletal damage, pectus excavatum with a Haller index of 53, cardiovascular damage including a bicuspid aortic valve and an 84 mm dilation of the ascending aorta, an ejection fraction (EF) of 40%, significant aortic and tricuspid valve insufficiency, leftward displacement of the cardiac chamber, and significant pulmonary involvement. The *FBN1* GV location was rs794728165 with a stop codon mutation type and reported severity of super pathogenicity. *SCN5A* GV are causally associated with Brugada syndrome, [92] long QT syndrome, cardiac conduction system dysfunction, and dilated cardiomyopathy, among others.

We found patients with *SDHA* GV encoding a major catalytic subunit of succinate-ubiquinone oxidoreductase, a mitochondrial respiratory chain complex, which could contribute to cardiovascular abnormalities such as mitral valve prolapse, aortic valve prolapses, and tricuspid regurgitation. Variants such as *SDHA* coexisted. Like other genes such as *DHB*, *SDHC*, and *SDHD*, *SDHA* is related to assembly factors, [93] which could explain the significant widespread musculoskeletal alterations.

We demonstrated this in a 15-year-old patient with Ehlers–Danlos syndrome who had coexisting SDHA and COL5A2 gene GV, where musculoskeletal and cardiovascular damage was severe; the patient also had bradyarrhythmia; in this case, the EDS classification was of the cardiac-vascular type which has been associated with COL5A2.

The interaction between MYBPC3 GV and other sarcomere genetic variants remains poorly understood [94]. The sarcomere, as the fundamental unit of skeletal and cardiac muscle contraction, constitutes more than 90% of the protein content of skeletal muscle and is vital for cardiac pumping and body movement [95].

In this study, the identification of other genes associated with the one that identifies each condition leads us to consider that there are patients with these conditions who have a wide variety of coexisting genes, such as those identified in this study. These variations may explain the significant phenotypic differences these patients present. Much has been discussed regarding the overlap of musculoskeletal and cardiovascular signs and alterations that exist among these diagnoses, which often cause confusion for physicians when trying to identify them within a specific diagnosis. We see that many of these patients meet criteria that classify them as having multiple myeloma (MM), and sometimes, in genetic screening, the reported gene is different from *FBN1*. Sometimes, *FBN1* is present but coexists with another gene that identifies a different condition.

In the MS, beyond the GV *FBN1* [62,96], various connective tissue disorders linked to different genes share overlapping mechanisms that could explain their diverse manifestations [97].

In the sub-analysis performed in this study, we observed that patients with coexisting genes other than the one that identifies their specific syndrome tend to have a lower left ventricular ejection fraction (LVEF). In these patients, cases with moderately and reduced left ventricular ejection fraction (LVEF) were identified, which was not observed in those without coexisting genes. While there is no statistically significant difference, this is understandable given the small number of cases without coexisting genes in this comparison. Therefore, future studies with a larger sample size and a specific focus on determining the average LVEF and its effect on systolic and diastolic dysfunction, as well as the impact of aortic dilation or valvular prolapse, will be necessary. However, the differences observed in this exploratory study support a detailed and prospective cardiovascular follow-up to evaluate the cardiovascular changes in each of these patients during follow-up, and a sequencing study that includes different hereditary genes to determine the reproducibility of these findings.

Understanding these mechanisms could lead to more effective genetic classification strategies and appropriate study and follow-up in the surveillance and development of targeted therapies to prevent life-threatening complications in both pediatric and adult patients.

It is important to highlight that the STRING-based network analyses presented here are purely computational approaches. They suggest possible structural and functional relevance based on aggregated data from multiple sources, but they do not demonstrate that these interactions are physiologically operative in our specific patients. Therefore, these findings should be considered hypothesis-generating, not mechanistic proof. Functional studies (e.g., protein expression, in vitro assays, or animal models) are required to validate the proposed interactions. Additionally, we recognize that the confirmation of NGS findings by Sanger sequencing would strengthen our results.

### Limitations

mRNA and protein expression analyses were not performed for the *FBN1* and *MYBPC3* genes and others with clinical relevance. While the in silico genetic, clinical, and structural data provided valuable information on the possible pathogenic mechanisms related to genes, and we were able to demonstrate, through next-generation sequencing, the coexistence of several genes in the same syndrome, which is related to complex clinical data, the lack of expression data limits our ability to fully understand the biological consequences of the identified variants. Additionally, we recognize that the confirmation of NGS findings by Sanger sequencing would strengthen our results. Future studies should include Sanger sequencing to confirm the reported variants and to assess segregation in affected families. Furthermore, the STRING-based network analysis presented in this study is hypothesis-generating and does not demonstrate physiologically operative interactions in our patients. Functional studies are required to test the hypotheses generated here. Family segregation analyses were not performed. Therefore, we cannot determine whether the identified genetic variants are inherited or de novo, nor can we assess the clinical phenotypes of carrier relatives.

## 5. Conclusions

The coexistence of genetic variants of multiple genes in various syndromic connective tissue disorders is associated with severe cardiovascular and musculoskeletal phenotypes. However, these findings are exploratory and hypothesis-generating; functional and familial studies are needed to determine whether these variants act as true genetic modifiers or contribute directly to damage mechanisms. The importance of these preliminary and exploratory findings underscores the need for multicenter studies incorporating functional assays and family analyses of the probands to determine whether the coexistence of the identified genetic variants could contribute to phenotypic heterogeneity or act as genetic modifiers in Marfan syndrome and other syndromes that clinically exhibit phenotypic and genotypic overlap.

## Figures and Tables

**Table 1:** Demographic data, comorbidities, frequency of Ghent criteria and VP of genes associated with connective tissue diseases.

Variable	MS *n* = 64	LDS *n* = 16	BHS *n* = 13	EDS *n* = 7	UCTD *n* = 35	*p*
Men *n* (%)	25 (39)	7 (44)	8 (61.5)	3 (43)	13 (37.1)	0.67
Age Mediana (Q1–Q3)	26 (20–35).	23 (21–41)	27 (13–33)	20 (15–28)	21 (17–31)	0.057
BMI Mediana (Q1–Q3)	22 (20–25)	21 (18–22)	22 (18–24)	20 (16–22)	20 (17–23)	0.14
Comorbidities *n* (%)						
Diabetes Mellitus	1 (1.5)	0 (0)	0 (0)	0 (0)	0 (0)	NS
High blood pressure	3 (4.5)	1(6.2)	0 (0)	1 (14.2)	1 (2.8)	0.46
Smoking	2 (3)	0 (0)	0 (0)	1(14.2)	0 (0)	0.90
Obesity	0 (0)	0 (0)	0 (0)	0 (0)	0 (0)	1
Dyslipidemias	3 (4.5)	0 (0)	0 (0)	0 (0)	0 (0)	0.73
Hypothyroidism	0 (0)	0 (0)	0 (0)	0 (0)	0 (0)	1
Ghent criteria MS *n* (%)						
Family history	43 (67.1)	5 (31.2)	6 (46.1)	0 (0)	8 (22.8)	0.0001
Ectopia Lentis	37 (57.8)	1 (6.2)	2 (15.3)	1 (14.2)	0 (0)	0.0001
Aortic dilation	51 (79.6)	7 (43.7)	2 (15.3)	2 (28.5)	3 (8.5)	0.0001
Systemic score	62 (96.8)	13 (81.2)	12 (92.3)	6 (85.7)	14 (40)	0.001
GV in *FBN1*	62 (96.8)	2 (12.5)	0 (0)	0 (0)	0 (0)	1
Genes associated with others CTD *n* (%)						
*TGFBR2*	2 (3.1)	13 (81.2)	1 (7.6)	0 (0)	0 (0)	0.0001
*TGFBR1*	0 (0)	3 (18.7)	0 (0)	0 (0)	0 (0)	0.0001
*FBN2*	6 (9.3)	3 (18.7)	13 (100)	0 (0)	0 (0)	0.0001
*COL3A1*	5 (7.8)	0 (0)	0 (0)	4 (57.2)	0 (0)	0.0001
*COL5A1*	3 (4.6)	0 (0)	0 (0)	1 (14.3)	2 (5.7)	0.54
*COL5A2*	0 (0)	0 (0)	0 (0)	2 (28.5)	1 (2.8)	0.002

MS = Marfan syndrome, LDS = Loeys–Dietz syndrome, BMI = Body mass index, CTD = connective tissue diseases, BHS = Beals–Hecht syndrome, EDS = Ehlers–Danlos syndrome, and UCTD = Undifferentiated connective tissue disease.

**Table 2:** Frequency of coexisting genes in each syndrome.

	Total *n* = 136	MS *n* = 64	LDS *n* = 16	BHS *n* = 13	EDS *n* = 7	UCTD *n* = 35
*FBN1*	64 (47)	62 (96.8)	2 (12.5)	0 (0)	0 (0)	0 (0)
*TGFBR2*	16 (11.7)	2 (3.1)	13 (81.2)	1 (7.6)	0 (0)	0 (0)
*TGFBR1*	3 (2.2)	0 (0)	3 (18.7)	0 (0)	0 (0)	0 (0)
*FBN2*	22 (16.1)	6 (9.3)	3 (18.7)	13 (81.2)	0 (0)	0 (0)
*COL3A1*	9 (6.6)	5 (7.8)	0 (0)	0 (0)	4 (57.1)	0 (0)
*COL5A1*	6 (3.6)	3 (4.6)	0 (0)	0 (0)	2 (28.5)	1 (2.8)
*COL5A2*	3 (2.2)	0 (0)	0 (0)	0 (0)	2 (28.5)	1 (2.8)
*MYBPC3*	25 (18.3)	8 (12.5)	6 (37.5)	2 (15.3)	0 (0)	9 (25.7)
*TTN*	15 (11)	7 (10.9)	1 (6.2)	2 (15.3)	1 (14.2)	4 (11.4)
*SDHA*	14 (10.2)	4 (6.2)	1 (6.2)	1 (7.6)	1 (14.2)	7 (20)
*TNNT2*	13 (9.5)	7 (10.9)	1 (6.2)	3 (23)	0 (0)	2 (5.7)
*AKAP9*	12 (8.8)	6 (9.3)	1 (6.2)	0 (0)	0 (0)	5 (14.2)
*MYPN1*	8 (5.8)	5 (7.8)	1 (6.2)	0 (0)	0 (0)	2 (5.7)
*DES*	6 (4.4)	3 (4.6)	0 (0)	0 (0)	0 (0)	3 (8.5)
*RYR2*	5 (3.6)	0 (0)	0 (0)	0 (0)	0 (0	5 (14.2)
*NOTCH1*	5 (3.6)	0 (0)	2 (12.5)	0 (0)	0 (0)	3 (8.5)
*PRDM16*	4 (2.9)	2 (3.1)	0 (0)	0 (0)	0 (0)	2 (5.7)
*RYR1*	4 (2.9)	2 (3.1)	0 (0)	0 (0)	0 (0)	2 (5.7)
*DSP*	4 (2.9)	1 (1.5)	0 (0)	2 (15.3)	0 (0)	1 (2.8)
*TTN-ASI*	3 (2.2)	0 (0)	1 (6.2)	1 (7.6)	0 (0)	1 (2.8)
*GCKR*	3 (2.2)	3 (4.6)	0 (0)	0 (0)	0 (0)	0 (0)
*HFE*	3 (2.2)	1 (1.5)	1 (6.2)	0 (0)	0 (0)	1 (2.8)
*MYLK*	2 (1.4)	0 (0)	1 (6.2)	0 (0)	1 (14.2)	0 (0)
*SOS1*	2 (1.4)	1 (1.5)	0 (0)	0 (0)	0 (0)	1 (2.2)
*NODAL*	2 (1.4)	2 (3.1)	0 (0)	0 (0)	0 (0)	0 (0)
*RBM20*	2 (1.4)	2 (3.1)	0 (0)	0 (0)	0 (0)	0 (0)
*TTR*	2 (2.4)	2 (3.1)	0 (0)	0 (0)	0 (0)	0 (0)
*LDLR*	2 (1.4)	2 (3.1)	0 (0)	0 (0)	0 (0)	0 (0)
*ANK2*	2 (1.4)	1 (1.5)	0 (0)	1 (7.6)	0 (0)	0 (0)
*SCN5A*	2 (1.4)	1 (1.5)	1 (6.2)	0 (0)	0 (0)	0 (0)
*ALMS1*	2 (1.4)	1 (1.5)	0 (0)	1 (7.6)	0 (0)	0 (0)
*HCN4*	2 (1.4)	1 (1.5)	0 (0)	0 (0)	0 (0)	1 (2.8)
*MYO6*	2 (1.4)	1 (1.5)	1 (6.2)	0 (0)	0 (0)	0 (0)
*LAMA2*	2 (1.4)	0 (0)	0 (0)	0 (0)	1 (14.2)	1 (2.8)
*ZBTB17*	2 (1.4)	0 (0)	0 (0)	0 (0)	0 (0)	2 (5.7)
*APOC4*	2 (1.4)	0 (0)	0 (0)	2 (15.3)	0 (0)	0 (0)
*APOC2*	2 (1.4)	0 (0)	0 (0)	2 (15.3)	0 (0)	0 (0)
*SNTA1*	1 (0.7)	0 (0)	0 (0)	0 (0)	0 (0)	0 (0)
*SALL4*	1 (0.7)	1 (1.5)	0 (0)	0 (0)	0 (0)	0 (0)
*MYH6*	1 (0.7)	1 (1.5)	0 (0)	0 (0)	0 (0)	0 (0)
*SELENON*	1 (0.7)	1 (1.5)	0 (0)	0 (0)	0 (0)	0 (0)
*DMD*	1 (0.7)	0 (0)	0 (0)	0 (0)	0 (0)	0 (0)
*SCL25A4*	1 (0.7)	1 (1.5)	0 (0)	0 (0)	0 (0)	0 (0)
*RAF1*	1 (0.7)	0 (0)	0 (0)	0 (0)	0 (0)	0 (0)
*DOLK*	1 (0.7)	1 (1.5)	0 (0)	0 (0)	0 (0)	0 (0)
*KCNE1*	1 (0.7)	1 (1.5)	0 (0)	0 (0)	0 (0)	0 (0)
*CREB3L3*	1 (0.7)	1 (1.5)	0 (0)	0 (0)	0 (0)	0 (0)
*KCNQ1*	1 (0.7)	1 (1.5)	0 (0)	0 (0)	0 (0)	0 (0)
*SCN3B*	1 (0.7)	0 (0)	1 (6.2)	0 (0)	0 (0)	0 (0)
*ABCG8*	1 (0.7)	0 (0)	1 (6.2)	0 (0)	0 (0)	0 (0)
*TRIM63*	1 (0.7)	0 (0)	1 (6.2)	0 (0)	0 (0)	0 (0)
*PRKAG2*	1 (0.7)	0 (0)	1 (6.2)	0 (0)	0 (0)	0 (0)
*SGCB*	1 (0.7)	0 (0)	0 (0)	0 (0)	0 (0)	1 (2.8)
*SMAD3*	1 (0.7)	0 (0)	0 (0)	0 (0)	0 (0)	1 (2.8)
*TRDN*	1 (0.7)	0 (0)	0 (0)	0 (0)	0 (0)	1 (2.8)
*GJA5*	1 (0.7)	0 (0)	0 (0)	0 (0)	0 (0)	1 (2.8)
*TPM1*	1 (0.7)	0 (0)	0 (0)	0 (0)	0 (0)	1 (2.8)
*HADHA*	1 (0.7)	0 (0)	0 (0)	0 (0)	0 (0)	1 (2.8)
*ZHX3*	1 (0.7)	0 (0)	0 (0)	0 (0)	0 (0)	1 (2.8)
*ACTN2*	1 (0.7)	0 (0)	0 (0)	0 (0)	0 (0)	1 (2.8)
*KCNH2*	1 (0.7)	0 (0)	0 (0)	0 (0)	0 (0)	1 (2.8)
*ACTA1*	1 (0.7)	0 (0)	0 (0)	0 (0)	0 (0)	1 (2.8)
*APOE*	1 (0.7)	0 (0)	0 (0)	1 (7.6)	0 (0)	0 (0)
*LDB3*	1 (0.7)	0 (0)	0 (0)	1 (7.6)	0 (0)	0 (0)
*APOB*	1 (0.7)	0 (0)	0 (0)	1 (7.6)	0 (0)	0 (0)
*PDLIM3*	1 (0.7)	0 (0)	0 (0)	1 (7.6)	0 (0)	0 (0)
*SCN2B*	1 (0.7)	0 (0)	0 (0)	1 (7.6)	0 (0)	0 (0)
*FKRP*	1 (0.7)	0 (0)	0 (0)	1 (7.6)	0 (0)	0 (0)
*TMPO*	1 (0.7)	0 (0)	0 (0)	1 (7.6)	0 (0)	0 (0)
*MIBI*	1 (0.7)	0 (0)	0 (0)	0 (0)	1 (14.2)	0 (0)
*HCN4A*	1 (0.7)	0 (0)	0 (0)	0 (0)	1 (14.2)	0 (0)
*JAG1*	1 (0.7)	0 (0)	0 (0)	0 (0)	1 (14.2)	0 (0)
*DNAJC19*	1 (0.7)	0 (0)	0 (0)	0 (0)	1 (14.2)	0 (0)
*TGFB3*	1 (0.7)	1 (1.5)	0 (0)	0 (0)	0 (0)	0 (0)

MS = Marfan syndrome. LDS = Loeys–Dietz syndrome. BHS = Beals–Hecht syndrome. UCTD = undifferentiated connective tissue disease.

**Table 3:** Frequency of Ghent criteria, type of genetic coexistence, and description of cardiovascular damage in patients with Marfan syndrome.

N	Sex	Age	Ghent Criteria					Total Ghent Criteria	Variant in Other Genes		LVEF	Surgery	Cardiovascular Damage
			FH	AD mm	EL	SS	*FBN1*						
**1**	F	18	+	35	+	+	+	5	*MYBPC3*	*MYPN*	46	---	AD, TR, AoVP, Posterior mitral valve blowing, dilation of the pulmonary trunk
**2**	F	41	-	44	+	+	+	4	*MYBPC3*	*TNNT2*	44	---	AD, compression of the RV
**3**	F	31	-	58	0	+	+	3	*MYBPC3*	*SALL4*	24	Ao Replace	AD, Eccentric LV hypertrophy, MR and TR
**4**	F	22	+	38	0	+	+	4	*MYBPC3*	*DES*	65	---	AD, intramyocardial fibrosis septal lineal
**5**	F	21	+	42	+	+	+	5	*MYBPC3*	*SOS1*	53	---	AD, MR, AoVP, TVP
**6**	F	40	+	55	+	+	+	5	*MYBPC3*	*TTN*	55	Cesarean B&B	AD, MR. Dissection in the immediate postpartum period
**7**	F	38	+	41	+	+	+	5	*MYBPC3*		45	---	AD, MVP, TVP
**8**	M	25	+	44	0	+	+	4	*MYBPC3*		26	B&B	AD, AoD, concentric hypertrophy of LV. TR
**9**	F	28	+	41	+	+	+	5	*TTN*		45	MVR	AoD, Barlow, syndrome, arrhythmogenic cardiomyopathy, MVP, LA dilation, sub mitral aneurysm, ventricular extrasystole
**10**	M	25	-	42	0	+	+	3	*TTN*		56	B&B	AD, MVP TVP, TR, Late septal enhancement with a non-ischemic pattern
**11**	M	31	-	42	0	+	+	3	*TTN*		26	---	AD, late non-ischemic strengthening, TVP, Ischemic cerebral in the middle brain
**12**	F	58	-	65	0	+	+	3	*TTN*	*SNTA1*	50	B&B	AD, Stanford A AoD, dilation of atria and LV remodeling, TR
**13**	M	26	+	48	+	+	+	5	*TTR*	*ALMS1*	62	B&B	AD, mild septal intramyocardial late enhancement. Severe PE
**14**	F	27	+	55	+	+	+	5	*TTR*		54	David II	AD, Oval foramen.
**15**	F	34	+	42	0	+	+	4	*COL5A1*	*JUP*	54	---	AD, Asymmetric septal, HCM, without left ventricular outflow tract obstruction. Mitral annulus dysfunction, MVP, TVP
**16**	F	15	+	+	0	+	+	4	*COL5A1*		ND	---	It is unknown, the patient stopped attending
**17**	F	20	-	+	0	+	+	3	*COL5A1*	*MYPN* *LDLR*	58	---	Mild MVP without insufficiency, mitral sub valvular aneurysm
**18**	M	28	+	41	+	+	+	5	*COL3A1*		55	---	AD, MVP both leaflets, died of a non-cardiac cause
**19**	M	41	+	40	+	+	+	5	*COL3A1*		51	MVR	AD, bi-atrial dilation MVP, bicuspid aortic valve
**20**	F	32	+	0	+	+	+	4	*COL3A1*	*MYPN*	56	---	MR, lack of coaptation of segments A2-P2 and A3-P3.
**21**	M	14	+	36	+	+	+	5	*COL3A1*	*PRDM16*	50	---	AD, Mild PI and AoR.
**22**	F	39	+	+	0	+	+	4	*COL3A1*	*GCKR* *PTDM16*	57	HDMB	Descending thoracic Ao, aneurysm with total abdominal dissection Stanford A De Bakey type III
**23**	F	32	+	39	+	+	+		*FBN2*		54	Cesarean	AD, 37 weeks pregnant, in labor Cesarean section
**24**	M	35	-	39	+	+	+	3	*FBN2*	*LAMA2*	57	---	AD, TR
**25**	M	24	+	+	-	+	+	4	*FBN2*	*MYH6*	58	---	It is unknown why he stopped going to the hospital
**26**	F	24	+	+	+	+	+	5	*FBN2*	*SELENON*	54	---	It is unknown why he stopped going to the hospital
**27**	F	59	+	+	+	+	+	5	*FBN2*	*LDLR*	59	---	It is unknown why he stopped going to the hospital
**28**	M	30	+	49	+	+	+	5	*FBN2*	*AKAP9*	52	---	AD, the patient stopped attending appointments.
**29**	F	35	+	60	+	+	+	5	*TGFBR2*	*DMD*	46	Cesarean B&B	AD, MVP, pregnancy of 34.3 weeks gestation, and the product was born without complications
**30**	1	7	+	+	-	+	+	4	*TGFBR2*		ND	---	It is unknown why he stopped going to the hospital
**31**	F	26	-	38	-	+	+	3	*SDHA*	*SLC25A4*	46	MVR	AD, MVP, Dilation of left heart chambers, fibrosis lateral wall, Sub valvular mitral aneurysm, arrhythmia required an ICD.
**32**	M	15	-	+	-	+	+	4	*SDHA*		41	---	AD, Z score 3.63, MVP, AoR. Mitral annulus dysfunction.
**33**	F	14	-	51	+	+	+	4	*SDHA*	*AKAP9*	55	---	AD, MVP, TVP, TR serious.
**34**	F	22	+	47	+	+	+	5	*SDHA*	*NODAL*	999	Ao replace	AD, AoR, MVP, MR
**35**	F	28	+	39	-	+	+	4	*RYR1*	*NODAL*	54	---	AD 39, MVP and Oval foramen
**36**	M	24	+	+	+	+	+	5	*AKAP9*		60	Ao replace	AoD 48 mm MVP, aortic valve bicuspid
**37**	F	14	-	36	+	+	+	4	*AKAP9*	*RBM20*	62	---	AD, Oval foramen
**38**	F	15	+	-	-	+	+	3	*AKAP9*	*RYR1*	48	NUSS	Pectum excavatum serious with extrinsic compression of the right ventricle.
**39**	F	45	-	-	+	+	+	3	*AKAP9*	*RYR1*	59	---	PDA tipo A de Krichenko, with bidirectional short circuit. MVP, TR mild
**40**	M	18	+	-	-	+	+	3	*MYPN*		53	---	Mild RV dilation. LV with sub mitral aneurysm.
**41**	M	19	-	38	-	-	+		*MYPN*		48	David F B&B	AD, Stanford B De Bakey IIIa AoD, Superior sinus venosus type atrial septal defect Anomalous inferior pulmonary vein drainage
**42**	F	37	-	-	-	+	+	2	*RAF1*		50	---	Late intramyocardial enhancement at junctional sites (non-ischemic), 4 mm, OS interatrial septal defect. TVP
**43**	F	4	+	+	+	+	+	5	*ANK2*		55	---	AD (Z score 3.9)
**44**	M	22	+	58	+	+	+	5	*DES*		58	David	AD, MVP
**45**	M	22	-	-	-	+	+	2	*PRDM16*		60	---	MVP
**46**	F	20	-	+	+	+	+	4	*LDLR*		ND	---	It is unknown why he stopped going to the hospital
**47**	M	36	+	84	+	+	+	5	*RBM20*	*LMF1*	52	B&B	AD, Cardiomegaly at the expense of the LV, PAH.
**48**	M	41	+	84	+	+	+	5	*SCN5A*		40	Patient RS	AD, PE, Haller index of 53, which causes displacement of the heart to the left and restriction of left bronchial compression, aortic valve bicuspid. Figure 4
**49**	F	9	+	35	-	+	+	4	*DOLK*	*TTN*	58	---	AD, TVP, MVP
**50**	M	20	+	-	+	-	+	3	*ALMS1*	*TTN*	ND	---	It is unknown why he stopped going to the hospital
**51**	F	31	+	39	-	+	+	4	*KCNE1*		56	---	AD
**52**	F	41	-	43	+	+	+	3	*GCKR*		65	---	AD, MR TR,
**53**	F	25	-	-	+	+	+	3	*GCRK*	*CREB3L3*	60		interatrial communication with occluder device
**54**	M	20	+	+	+	+	+	5	*KCNQ1*	*HCN4*	57	---	AD Z score 3.3, MVP, PE, Haller index of 4.4 with restricted diastolic ventricular movement
**55**	F	10	+	-	-	+	+	3	*HFE*	*DSP* *DES*	57	---	Compression of the free wall of the right ventricle in the middle and apical thirds due to pectus excavatum. Haller index of 5.8
**56**	F	27	+	38	+	+	+	5	----	------	59	---	AD.
**57**	M	34	+	36	+	+	+	5	----	------	57	---	AD, TR, MVP anterior y septal.
**58**	M	15	-	+	-	+	+	3	----	------	59	---	AD Score 2.2, dilation coronary artery trunk and CHD (left coronary artery to LV fistula)
**59**	M	37	+	41	+	+	+	5	-----	------	50	---	AD, MVP and MR serious, TR
**60**	M	45	-	52	+	+	+	4			58	B&B	AD, AoD Stanford A, DeBakey 1 involves supara-aortic trunks, extending to the left common iliac artery. The left renal and inferior mesenteric arteries emerge from the false lumen.
**61**	F	45	+	41	-	+	+	4	-----	-----	54	---	AD, billowing mitral valve, MR
**62**	M	14	+	0	+	+	+	5	-----	-----	56	---	Sin dilatación aortica sin compromiso valvular
**63**	F	32	-	+	-	+	+	3	----	----	ND	---	It is unknown why he stopped going to the hospital
**64**	1	22	+	35	0	+	+		*MYO6*		56	---	AD, Bivalve aorta due to fusion of the right and left coronary valves AoR, MVP Disjunction of the mitral and tricuspid annulus.

FH = family history, AD = aortic dilation, AoD = aortic dissection, EL = ectopia lentis, and SS = systemic score. TR = tricuspid regurgitation, PI = pulmonary insufficiency, OS = ostium secundum, PAH = pulmonary arterial hypertension, ICD = implantable cardioverter defibrillators, PE = pectux excavatim, MVP = aortic valve prolapse, RV = roght ventricle, LV = left ventricle, MR = mitral regurgitation, MV = mitral valve, TVP = tricuspid valve prolapse, CHD = congenital heart disease, B&B S = Bentall and Bono surgery and cesarean, LA = left atrio, AoR = aortic regurgitation, PDA = patent ductus arteriosus, RV = right ventricle, MVR = mitral valve replacement, HCM = hypertrophic cardiomyopathy, ND = not done, HDBM = hospital discharge for maximum benefit, DavidF = David failure, and Patient RS = the patient refuses surgery.

**Table 4:** Frequency of cardiovascular damage in metabolic syndrome and other connective tissue disorders.

Variable	MS *n* = 64	LDS *n* = 16	BHS *n* = 13	EDS *n* = 7	UCTD *n* = 35	*p*
	Median (Min–Max)f					
LVEF	56 (24–66)	51 (34–60)	56 (50–64)	61 (60–66)	62 (41–70)	0.01
Mitral valve prolapse	24 (37.5)	1 (6.2)	3 (23)	1 (14.2)	3 (8.5)	0.18
Aortic valve prolapse	2 (3.1)	1 (6.2)	1 (7.6)	0 (0)	0 (0)	0.45
Tricuspid valve prolapse	9 (14)	2 (12.5)	0 (0)	0 (0)	1 (2.8)	0.40
Mitral regurgitation	10 (15.6)	5 (31.2)	4 (30.7)	0 (0)	4 (11.4)	0.02
Aortic regurgitation	4 (6.2)	1 (6.2)	0 (0)	1 (14.2)	1 (2.8)	0.49
Tricuspid regurgitation	8 (12.5)	4 (25)	4 (30.7)	1 (14.2)	2 (5.7)	0.02
Pulmonary insufficiency	1 (1.5)	2 (12.5)	3 (23)	1 (14.2)	0 (0)	0.001
Arrhythmogenic CM	1 (1.5)	0 (0)	0 (0)	0 (0)	0 (0)	0.89
Pulmonary artery dilation	2 (3.1)	2 (12.5)	2 (15.3)	1 (14.2)	2 (5.7)	0.09
Aortic diameters	Median (Min–Max)					
Aortic valve plane (mm)	26 (15–84)	26 (14–34)	23 (19–27)	21 (15–28)	19 (14–26)	0.001
Valsalva sinus (mm)	38 (13–84)	36 (22–48)	31 (19–38)	28 (22–48)	25 (15–33)	0.001
Sinotubular junction (mm)	27 (14–63)	25 (16–36)	24 (17–29)	23 (17–30)	21 (12–32)	0.04
Ascending aorta (mm)	26 (15–58)	27 (19–40)	23 (18–26)	21 (15–29)	22 (13–42)	0.10
	n (%)					
Congenital heart disease	10 (15.6)	3 (18.7)	0 (0)	0 (0)	6 17.1)	0.09
Type of CHD						
Bicuspid aorta	5 (7.8)	1 (6.2)	0 (0)	0 (0)	4 (11.4)	0.46
Bicuspid aorta, IVC and PDA	0 (0)	0 (0)	0 (0)	0 (0)	2 (5.7)	0.70
Coronary artery to LV	1 (1.5)	0 (0)	0 (0)	0 (0)	0 (0)	0.89
Inter-atrial communications	1 (1.5)	1 (6.2)	0 (0)	0 (0)	0 (0)	0.45
OS and IV communication	1 (1.5)	0 (0)	0 (0)	0 (0)	0 (0)	0.89
patent ductus arteriosus	1 (1.5)	0 (0)	0 (0)	0 (0)	0 (0)	0.89
Anomalous vs. and AD	1 (1.5)	0 (0)	0 (0)	0 (0)	0 (0)	0.89
PDA and IVC	0 (0)	1 (6.2)	0 (0)	0 (0)	0 (0)	0.65
Hypoplastic aortic arch	0 (0)	0 (0)	0 (0)	0 (0)	1 (2.8)	0.70
Septal fibrosis	4 (6.2)	0 (0)	1 (7.6)	0 (0)	0 (0)	0.86
Ventricular dysfunction	19 (29.6)	3 (18.8)	2 (15.4)	1 (14.3)	3 (8.6)	0.17
Aortic dissection	6 (9.4)	1 (6.3)	0 (0)	1 (14.3)	0 (0)	0.36
Surgery	20 (31)	4 (25)	2 (15.3)	0 (0)	3 (8.5)	0.25
Bental y DeBono	6 (9.3)	1 (6.3)	0 (0)	0 (0)	1 (2.8)	0.18
Cesarean and BB	2 (3.1)	0 (0)	0 (0)	0 (0)	0 (0)	0.79
Florida Sleeve	1 (1.5)	2 (12.5)	0 (0)	0 (0)	0 (0)	0.60
David	3 (4.6)	0 (0)	0 (0)	0 (0)	0 (0)	0.69
Asc Ao A	1 (1.1)	0 (0)	0 (0)	0 (0)	0 (0)	0.89
aortic valve replacement	2 (3.1)	0 (0)	0 (0)	0 (0)	0 (0)	0.60
MVR	1 (1.5)	1 (6.2)	1 (7.6)	0 (0)	0 (0)	0.45
Coartectomia	0 (0)	1 (6.2)	0 (0)	0 (0)	0 (0)	0.65
MVR and TVR	1 (1.1)	0 (0)	0 (0)	0 (0)	0 (0)	0.89
AoVR, MVR yTVR	0 (0)	0 (0)	0 (0)	0 (0)	1 (2.8)	0.70
NUSS technique	2 (3.1)	0 (0)	1 (7.6)	0 (0)	1 (2.8)	0.46
Cesarean	1 (1.5)	0 (0)	0 (0)	0 (0)	0 (0)	0.89
Interventional treatment	1 (1.5)	2 (12.5)	0 (0)	2 (0)	3 (8.5)	0.75
CIA	1 (1.5)	1 (6.3)	0 (0)	0 (0)	0 (0)	0.89
Ablation	0 (0)	0 (0)	0 (0)	1 (14.3)	1 (2.8)	0.40
Abdominal aortic prosthesis	0 (0)	0 (0)	0 (0)	1 (14.3)	0 (0)	0.74
Closure of PDA	0 (0)	0 (0)	0 (0)	0 (0)	2 (5.7)	0.60
TAVI	0 (0)	1 (6.2)	0 (0)	0 (0)	0 (0)	0.58

LVEF = left ventricular ejection fraction, CM = cardiomyopathy, CHD = congenital heart disease, LV = left ventricle. AD = atrial defect, VS = venous sinus, MVR = mitral valve replacement, TVR = tricuspid valve replacement, AoVR = aortic valve replacement, Asc Ao A = ascending aortic aneurysmectomy, BB = Bentall and DeBono, CIA = closure of interatrial communication, TAVI = transcatheter aortic valve implantation, MS = Marfan syndrome, LDS = Loeys–Dietz syndrome, BHS = Beals–Hecht syndrome, EDS = Ehlers–Danlos syndrome, UCTD = undifferentiated connective tissue disease, IVC = interventricular communication, OS = ostium secundum, PDA = patent ductus arteriosus, and N/A: No Aplica. Test Fisher, X2, y U Mann–Whitney. Tukey *p*-adjusted values, SD = standard deviation, mm milímetros s. OR = odds ratio, X = media, CI = confidence interval.

**Table 5:** Sub-analysis of cardiovascular status using left ventricular ejection fraction and aortic diameters in patients who tested positive for *MYBPC3*, and what was analyzed with an age- and sex-matched group that was negative for MYBPC3.

	All Groups of Disease		*p*
	*MYBPC3*+ n = 17	*MYBPC3*− n = 17	
	Median (Min–Max)	Median (Min–Max)	
Left ventricular ejection fraction	51 (24–60)	55 (50–70)	0.04
Preserved LVEF	10 (59)	17 (100)	0.007
Moderately reduced LVEF	5 (29.4)	0 (0)	0.04
Reduced LVEF	2 (11.7)	0 (0)	0.02
Valvular plane aortic annulus	24 (14–35)	24 (17–34)	0.58
Valsalva sinus	38 (16–60)	33 (16–51)	0.32
Sinotubular junction	28.5 (15–48)	25 (15–32)	0.08
Ascending aorta	26 (14–42)	24 (13–40)	0.08
	Marfan syndrome		*p*
	*MYBPC3*+ *n* = 8	*MYBPC3*− *n* = 8	
	Median (Min–Max)	Median (Min–Max)	
Left ventricular ejection fraction	47 (24–56)	54 (50–60)	0.10
Preserved LVEF	4 (50)	8 (100)	0.07
Moderately reduced LVEF	2 (25)	0 (0)	0.46
Reduced LVEF	2 (25)	0 (0)	0.46
Valvular plane aortic annulus	24 (17–28)	27 (21–34)	0.19
Valsalva sinus	41 (30–60)	36 (26–51)	0.27
Sinotubular junction	28 (24–48)	26 (20–30)	0.16
Ascending aorta	26 (24–32)	24 (20–28)	0.13
	Loeys–Dietz		
	*MYBPC3*+ *n* = 4	*MYBPC3*− *n* = 4	
Left ventricular ejection fraction	47 (45–60)	54 (53–55)	0.24
Preserved LVEF	1 (25)	4 (100)	1
Moderately reduced LVEF	3 (75)	0 (0)	0.14
Reduced LVEF	0 (0)	0 (0)	N/A
Valvular plane aortic annulus	32 (14–35)	29 (22–34)	0.72
Valsalva sinus	42 (22–49)	36 (30–45)	0.72
Sinotubular junction	34 (16–36)	28 (22–32)	0.47
Ascending aorta	36 (21–40)	26(21–34)	0.37
	Undifferentiated connective tissue disease		
	*MYBPC3*+ *n* = 5	*MYBPC3*− *n* = 5	
Left ventricular ejection fraction	60 (53–60)	64 (55–70)	0.19
Preserved LVEF	5 (100)	5 (100)	1
Moderately reduced LVEF	0 (0)	0 (0)	N/A
Reduced LVEF	0 (0)	0 (0)	N/A
Valvular plane aortic annulus	20 (19–26)	19 (17–22)	0.41
Valsalva sinus	25 (16–33)	20 (16–33)	0.90
Sinotubular junction	28 (15–32)	17 (15–31)	0.41
Ascending aorta	31 (14–42)	17 (13–40)	0.41

**Table 6:** Types of surgery performed on matched patients in relation to the presence of the *MYBPC3* genetic variant.

Marfan Syndrome	*MYBPC3*+ *n* = 8	*MYBPC3*− *n* = 8	Total *n* = 16
Without surgery	4 (50)	6 (75)	10 (62.5)
Bentall and Bono	2 (25)	1 (12.5)	3 (19)
Cesárea + Bentall and Bono	1 (12.5)	0 (0)	1 (6.2)
Aortic valve replacement	1 (12.5)	0 (0)	1 (6.2)
Tirone David	0 (0)	1 (0)	1 (6.2)
Loeys–Dietz Syndrome	*MYBPC3*+ *n* = 4	*MYBPC3*− *n* = 4	Total *n*= 8
Without surgery	0 (0)	1 (25)	1 (12.5)
Bentall and Bono	1 (25)	1 (25)	2 (25)
Florida sleeve	1 (25)	1 (25)	2 (25)
Aortic valve replacement	1 (25)	0 (0)	1 (12.5)
Closure of atrial septal defect	1 (25)	0 (0)	1 (12.5)
Coartectomy	0 (0)	1 (25)	1 (12.5)
Undifferentiated connective tissue disease	*MYBPC3*+ *n* = 5	*MYBPC3*− *n* = 5	Total *n* = 10
Without surgery	3 (60)	5 (100)	8 (80)
Closure of the ductus arteriosus	1 (20)	0 (0)	1 (10)
NUSS surgery	1 (20)	0 (0)	1 (10)
*Beal’s Hecht syndrome*	*MYBPC3+* *n* = 1	*MYBPC3*− *n* = 1	Total *n* = 2
Mitral valve replacement	1 (100)	0 (0)	1 (50)

## Data Availability

Due to confidentiality agreements, the data underlying this study are not publicly available. Access to the data can be requested through mesoto50@hotmail.com following their confidentiality protocols.

## Supplementary Materials

The following supporting information can be downloaded at https://www.mdpi.com/article/10.3390/cells15111001/s1, File S1. Supplementary Dataset. Video S1. Case 1. Video S2. Case 2. Table S1. Frequency of Ghent criteria, type of genetic coexistence, and description of cardiovascular damage in patients with Loeys Dietz, Beals Hecht and Ehlers Danlos. Table S2. Frequency of Ghent criteria, type of genetic coexistence, and description of cardiovascular damage in patients with Undifferentiated connective tissue disease (UCT).

## Abbreviations

The following abbreviations are used in this manuscript:

MS	Marfan syndrome
LDS	Loeys–Ditz syndrome
BHS	Beals–Hecht syndrome
EDS	Ehlers–Danloas syndrome
UCTD	Undifferentiated connective tissue disease
GV	Genetic variants
NGS	Next-generation sequencing
AD	Aortic dilation
EL	Ectopia lentis
PPI	Protein–protein interaction
VSD	Ventricular septum defect
OS	Ostium secundum
CHD	Congenital heart disease
LVEF	Left ventricular ejection fraction
LV	Left ventricular
SGL	Strain global longitudinal
CTA	Computed tomography angiography
ECM	Extracellular matrix
VUS	Uncertain significance
ICD	Implantable cardioverter defibrillators
