# Supplementary material for: Cardiovascular and Clinical Manifestations of Marfan Syndrome and Other Inherited Connective Tissue Disorders with Coexisting Genetic Variants
Source: Cells. 2026 May 29;15(11):1001. doi: 10.3390/cells15111001 (PMC13256586; doi:10.3390/cells15111001)
Supplement: Supplementary file 1 [file cells-15-01001-s001.zip › TABLE S1.pdf]

Table S1 Frequency of Ghent criteria, type of genetic coexistence, and description of cardiovascular damage in patients with Loeys Dietz, Beals Hecht and Ehlers Danlos

No	Sex	Age	Ghent criteria					Total Ghent Criteria	TGFBR	VP in other enes	LVEF	Surgery	Cardiovascular damage
**Loeys Dietz Syndrome**													
			FH	DA	EL	SS	FBN						
**1**	M	18	-	-	-	+	-	1	*TGFBR2*	*MYBPC3* *MY06*	48	---	Bicuspid aortic valve with double lesion. Stenosis at the Sino tubular junction. Celiac trunk with angulation at its origin and dilation prior to its bifurcation. Subaortic aneurysm. TR. Anomalous origin of the left subclavian artery. Hypertelorism
**2**	F	50	+	-	-	+	-	2	*TGFBR2*	*MYBPC3* *MYLK2*	ND	---	Hypertelorism. The patient went to the Social Security Hospital for further evaluation of his condition, so we do not know his imaging studies or their progress.
**3**	M	23	-	-	-	+	-	1	*TGFBR2*	*MYBPC3* *SCN3B*	ND	---	Additional clinical data, hypertelorism, bifid uvula .It is unknown why he stopped going to the hospital
**4**	M	22	-	-	-	+	-	1	*TGFBR2*	*MYBPC3* *NOCHT1*	60	Closure of atrial septal defect	Additional clinical data: elongated and wrinkled ears, hypertelorism. Closure of the atrial septal defect with Amplatzer procedure without residual shunts. MR.
**5**	M	48	0	+	0	+	-	2	*TGFBR2*	*MYBPC3* *HFE*	47	Florida Sleeve Pulmonary artery replacement	Ao D 49 mm, Right and Right atrium, pulmonary artery dissection, dilation of the right heart chambers, hypertrophy of the right ventricular free wall, and pulmonary and TR. Extensive anteroseptal and inferoseptal late intramyocardial enhancement at the junction sites of the LV with RV, basal and middle thirds
**6**	F	22	+	+	-	+	-	3	*TGFBR2*	*MYBPC3* *NOCHT1*	55	---	AoD Z score 2.52. Additional data Hypertelorism, pes cavus, pectus carinatum milia in skin
**7**	M	21	-	+	-	+	-	2	*TGFBR1*	*FBN2* *TTN-AS1*	44	Florida Sleeve	AoD 45 mm. Additional data, Vertebral arteries with a tortuous course, dilation of the pulmonary artery trunk, left ventricle with concentric hypertrophy.
**8**	F	4	-	-	-	+	-	1	*TGFBR2*	*FBN2* *TTN*	ND	---	Additional clinical data, hypertelorism, uvula bifida It is unknown why he stopped going to the hospital
**9**	M	35	-	-	-	+	-	1	*TGFBR2*	*FBN2* *ABCG8*	ND	---	It is unknown why he stopped going to the hospital Hypertelorism
**10**	F	21	+	-	-	+	+	2	*TGFBR1*	*TRIM63*	ND	---	It is unknown why he stopped going to the hospital
**11**	F	45	-	-	-	+	+	1	*TGFBR1*	*MYPN*	34		Dilated cardiomyopathy with enhancement of non-ischemic pattern. Aneurysmal interatrial septum with

													bulging into the right atrium, without evidence of shunting.
**12**	1	43	-	+	-	+	-	2	*TGFBR2*	*PRKAG2*	40	B & B	AoD 55 mm,Barlow's disease MR, TR, paroxysmal atrial tachycardia. Additional data Arachnoidocela sillar encephalomalacia
**13**	1	27	+	+	-	-	-	2	*TGFBR1*	*SCN5a*	55	-----	AoD 40 mm, It is unknown why he stopped going to the hospital.
**14**	F	38	-	-	-	+	-	1	*TGFBR2*	*SDHA*	ND	----	She missed her MRI evaluation appointment and could no longer be located; she was from out of town. Additional data: uvula bifida and hypertelorism
**15**	M	22	-	-	-	+	-	1	*TGFBR2*	*MYBPC3*	ND	---	He did not return and was not evaluated by imaging studies. Additional information indicated that the patient had hypertelorism, cavus foot, and milia.
**16**	F	15	-	+	-	-	-	1	*TGFBR2*		40	Coartectomy closure of VSD	AoD 45 mm, Patent ductus arteriosus and ventricular septal defect Additional data: hypertelorism, cavus foot, tortuous arteries

Beals Hecht syndrome

**No**	Sex	Age	Ghent criteria					Total Ghent Criteria	*FBN2*	*VP in other genes*	LVEF %	Surgery/Intervent on	Cardiovascular damage
			FH	DA	EL	SS	FBN						
**1**	M	35	+	-	-	+	-	2	*FBN2*	*MYBPC3 SDHA*	51	mitral plasty	AoD 33 mm, eccentric LV hypertrophy, MVP Additional clinical data: Contractural arachnodactyly in hands and feet
**2**	M	35	+	-	-	+	-	2	*FBN2*	*MYBPC3 TGFBR2*	ND	---	The patient did not continue with his care at the hospital and decided to go to his social security care center.
**3**	M	39	+	-	+	+	-	3	*FBN2*	*TTN APOE*	68	mitral valve replacement and NUSS surgery	AoD 38 mm, severe MVP, submitral aneurysm, severe TR MRAdditional data: contractural arachnodactyly in feet and wrinkled ears
**4**	M	8	-	+	+	+	-	3	*FBN2*	*TTN*	57	---	AoD Z score 4.22,MR, TR pulmonary reguirgitation.dilation of pulmonary branches additional dates: contractural arachnodactylywrinkled ear
**5**	F	32	-	+	-	+	-	2	*FBN2*	*LDB3*	62	---	Extrinsic compression of the free wall of the right ventricle. Interatrial septal aneurysm MR TR. Contractural arachnodactyly
**6**	M	27	+	-	-	+	-	2	*FBN2*	*APOB*	62	---	Mitral valve prolapse without insufficiency. Pectus excavatum causing asymmetry of the left hemithorax and diastolic compression of the right ventricle. Contractural arachnodactyly

**7**	F	16	-	-	-	+	-	1	*FBN2*	*TTN-AS1*	58	posterolateral ablation	Wolff-Parkinson-White syndrome with left posterolateral ablation successes
**8**	M	31	-	-	-	+	-	1	*FBN2*	*APOC4-APOC2*	58	---	The patient presented with lymphadenopathy in the neck and was referred to another institution for further evaluation, where he was diagnosed with lymphoma. He did not return to our institution. Additional data: wrinkled ear, arachnodactyly, contractural
**9**	F	30	-	-	-	+	-	1	*FBN2*	*ANK2*<br>*PDLIM3*	ND	---	The patient did not return for his admission process and continuation of studies at the institution. wrinkled ear
**10**	2	12	-	-	-	+	-	1	*FBN2*	*DSP*<br>*SCN2B*	ND	-----	The patient did not return for his admission process and continuation of studies at the institution. arachnodactyly, contractural
**11**	2	16	+	-	-	+	-	2	*FBN2*	*FKRP*	ND	----	The family member decided to treat the patient privately.
**12**	1	20	-	-	-	+	-	1	*FBN2*	*ALMS1*	ND		The patient did not return for his admission process and continuation of studies at the institution elf ear, arachnodactyly, contractural
**13**	2	14	+	-	-	-		1	*FBN2*	*APOC4-APOC2*<br>*TMPO*	50	---	Minimal late non-ischemic pattern enhancement at RV-LV junction sites. Concentric remodeling of the LV, TR PR. Additional data: wrinkled earlobes and arachnodactyly in feet

Ehlers Danlos

**No**	Sex	Age	Ghent criteria					Total Ghent Criteria	*COL3A1/ COL5A1*	*VP in other genes*	LVEF %	Surgery/Intervent on	Cardiovascular damage
			FH	DA	EL	SS	FBN						
**1**	F	15	-	-	-	+	-	1	*COL5A2*	*MIB1*	ND	----	The patient did not return for his admission process and continuation of studies at the institution. Additional information: Hyperelasticity was found in it
**2**	M	10	-	+	+	+	-	3	*COL5A2*	*SDHA*<br>*HCN4A*	60	Plasty mitral valve ,aortic valve change, Successful ablation by FA	AoD Z Score 2.85 pulmonary artery aneurysm 54 mm, hypertrophic right ventricle, coronary ectasia of the right coronary artery, left anterior descending artery. Additional data: significant severe chest deformity and scoliosis
**3**	F	20	-	-	-	+	-	1	*COL5A1*	*JAG1*	57		Without aortic dilation and without valvular disease, he has significant musculoskeletal hyperlaxity and deformity.

**4**	M	32	-	+	-	-	-	1	*COL3A1*	*MYLK*	60	Abdominal aortic implantation and 3 stents connected to the left femoral artery. Supracondylar amputation	Abdominal aneurysm, dissection of left iliac artery, compartment syndrome and Rhabdomyolysis. Additional date elf ear, contractural arachnodactyly
**5**	M	19	-	-	-	+	-	1	*COL3A1*	*APOE*	66	----	Without aortic dilation and without valvular disease. Right paravertebral nodular lesion of probable neurogenic origin.
**6**	F	23	-	-	-	+	-	1	*COL3A1*	*DNAJC19* *LAMA2*	ND	---	The patient did not return for his admission process and continuation of studies
**7**	F	28	-	-	-	+	-	1	*COL3A1*	*TTN*	ND	---	The patient did not return for his admission process and continuation of studies
