# Supplementary material for: Cardiovascular and Clinical Manifestations of Marfan Syndrome and Other Inherited Connective Tissue Disorders with Coexisting Genetic Variants
Source: Cells. 2026 May 29;15(11):1001. doi: 10.3390/cells15111001 (PMC13256586; doi:10.3390/cells15111001)
Supplement: Supplementary file 1 [file cells-15-01001-s001.zip › TABLE S2.pdf]

Table S2 Frequency of Ghent criteria, type of genetic coexistence, and description of cardiovascular damage in patients with Undifferentiated connective tissue disease (UCT)

No	Sex	Age	Ghent criteria					Total Ghent Criteria	UCTD with VP in other genes		LVEF	Surgery	Cardiovascular damage
			FH	DA	EL	SS	FBN1+						
**1**	F	46	-	-	-	-	-	1	*MYBPC3*	*LAMA2*	60	---	Details about her are unknown; after the first evaluation, she did not return because she decided to seek treatment at a private institution.
**2**	F	54	-	-	-	+	-	0	*MYBPC3*	*SDHA* *SMAD3*	ND	----	Bicuspid aortic valve
**3**	F	20	-	-	-	+	-	1	*MYBPC3*	*SDHA*	60	---	no heart disease, no valvular heart disease, and no rhythm disorders, she had positive scores >7 points on the Ghent criteria SS, she complained of muscle pain
**4**	F	48	-	-	-	-	-	1	*MYBPC3*	*TRDN, HFE*	65	---	AD 39 mm TR
**5**	M	23	1	0	0	+	-	2	*MYBPC3*	*DES, DSP*	60	---	normal heart chambers without valvular disease
**6**	F	50	0	0	0	0		0	*MYBPC3*	*DES*	ND	---	She did not attend to continue his studies
**7**	M	40	0	0	0	0		0	*MYBPC3*	*SOS1*	ND	---	He did not attend to continue his studies
**8**	F	45	0	0	0	+		1	*MYBPC3*		54		AoD 42 mm, Bicuspid aortic valve
**9**	M	17	0	0	0	+		1	*MYBPC3*		53	---	AoD Z score 3.3 , Bicuspid aortic valve
**10**	M	18	0	0	0	+		1	*TTN*		ND	---	He did not attend to continue his studies
**11**	1	19	0	+	0	0		1	*TTN*	*SDHA*		closure ductus arteriosus	AD. Bicuspid aortic valve, patent ductus arteriosus
**12**	2	23	0	0	0	0		0	*TTN*	*ZBTB17*	46	---	Additional data, sudden palpitations, and normal Holter report The patient did not return for another cardiology appointment.
**13**	2	27	0	0	0	+		1	*TTN2*		ND	---	He did not attend to continue his studies

**14**	1	33	1	0	0	0	1	*TTN-AS1*		60	---	Normal geometry and cardiac chamber diameters, no valvular heart disease
**15**	2	15	0	+	0	0	1	*MYPN*	*ZBTB17*	59	---	AD,Z score 2.27 AoR, bicuspid aortic valve
**16**	2	55	0	0	0	0	0	*AKAP9*	*DES*	ND	---	He did not attend to continue his studies
**17**	1	20	0	0	0	0	1	*AKAP9*				She did not attend to continue his studies
**18**	2	15	0	0	0	+	1	*AKAP9*	*PRDM16*	54	---	AD,Z score 2.10 ventricular septal defect, patent ductus arteriosus, MVP, outflow tract obstruction
**19**	1	31	+	0	0	0	1	*AKAP9*	*GJA5*	50	---	Left ventricular dilation, eccentric hypertrophy of the left ventricle, generalized hypo kinesis of the left ventricle. Significant mitral regurgitation, bicuspid aortic valve
**20**	1	23	0	0	0	0	1	*AKAP9*	*COL5A2*	61	---	Normal echocardiographic study.<br>As an additional detail, she has had dysautonomia.
**21**	1	20	0	0	0	0	0	*SDHA*				She did not attend to continue his studies
**22**	1	16	0	0	0	0	0	*SDHA*		ND	---	She did not attend to continue his studies
**23**	1	18	0	0	0	0	0	*SDHA*	*RYR2*	ND	---	She did not attend to continue his studies
**24**	1	22	0	0	0	+	1	*RYR2*		ND	---	She had dyspnea but did not attend to continue his studies
**25**	1	20	0	0	0	0	0	*RYR2*	*HCN4 NOTCH1*	ND	---	She did not attend to continue his studies
**26**	1		0	0	0	+	1	*RYR2*	*SDHA TPM1*	35	Aortic arch replacement, supra-aortic trunk reimplantation, and B&B	Hypoplasia aortic arch, stenosis at the origin of the brachiocephalic trunk and left common carotid artery, severe MR and moderate TR, dilation of the pulmonary artery trunk.
**27**	2	22	1	0	0	0	1	*RYR2*	*COL5A1*	ND	---	He did not attend to continue his studies

**28**	1	18	0	+	0	0	1	*RYR1*		66	---	MVP, MR
**29**	2	20	+	0	0	+	2	*NOTCH1*		64	---	Normal geometry cardiac chamber diameters, no valvular heart disease Pectus excavatum left ventricular restriction,.
**30**	1	21	0	0	0	+	1	*NOTCH1*	*TTN* *HADHA*	63	---	Without heart or valve damage
**31**	1	20	0	0	0	+	1	*ZHX3*		61	---	Without heart or valve damage
**32**	1	15	0	0	0	+	1	*ACTN2*		68	---	MVP
**33**	2	14	+	0	0	0	1	*KCNH2*		ND	---	He did not attend to continue his studies
**34**	1	13	+	0	0	0	1	*PRMD16*	*ACTA1*			Normal geometry cardiac chamber diameters, no valvular heart disease
**35**	2	31	0	0	0	+	1	*TNNT2*		59	---	Severe pectus excavatum causing compression of the right ventricular free wall; DVT, minimal late enhancement with a non-ischemic pattern; dilation of the pulmonary artery trunk.

FH= Family history, AD=Aortic dilation, EL=Ectopia Lentis, SS= Systemic Score. TR = Tricuspid regurgitation , AoVP = aortic valve prolapse, RV0 right ventricle, LV= left ventricle, MR= Mitral regurgitation, TVP = tricuspid valve prolapse, B&B S= Bentall and Bono surgery and cesarean, LA left atrium, AoR= aortic regurgitation, PDA= Patent ductus arteriosus, RV Right ventricle, MVR= mitral valve replacement, ND= Not done, HDBM= Hospital discharge for maximum benefit, David F= David failure, Patient RS= The patient refuses surgery
