# Supplementary figures and images for: Cardiovascular and Clinical Manifestations of Marfan Syndrome and Other Inherited Connective Tissue Disorders with Coexisting Genetic Variants
Source: Cells. 2026 May 29;15(11):1001. doi: 10.3390/cells15111001 (PMC13256586; doi:10.3390/cells15111001)

## Slide 1
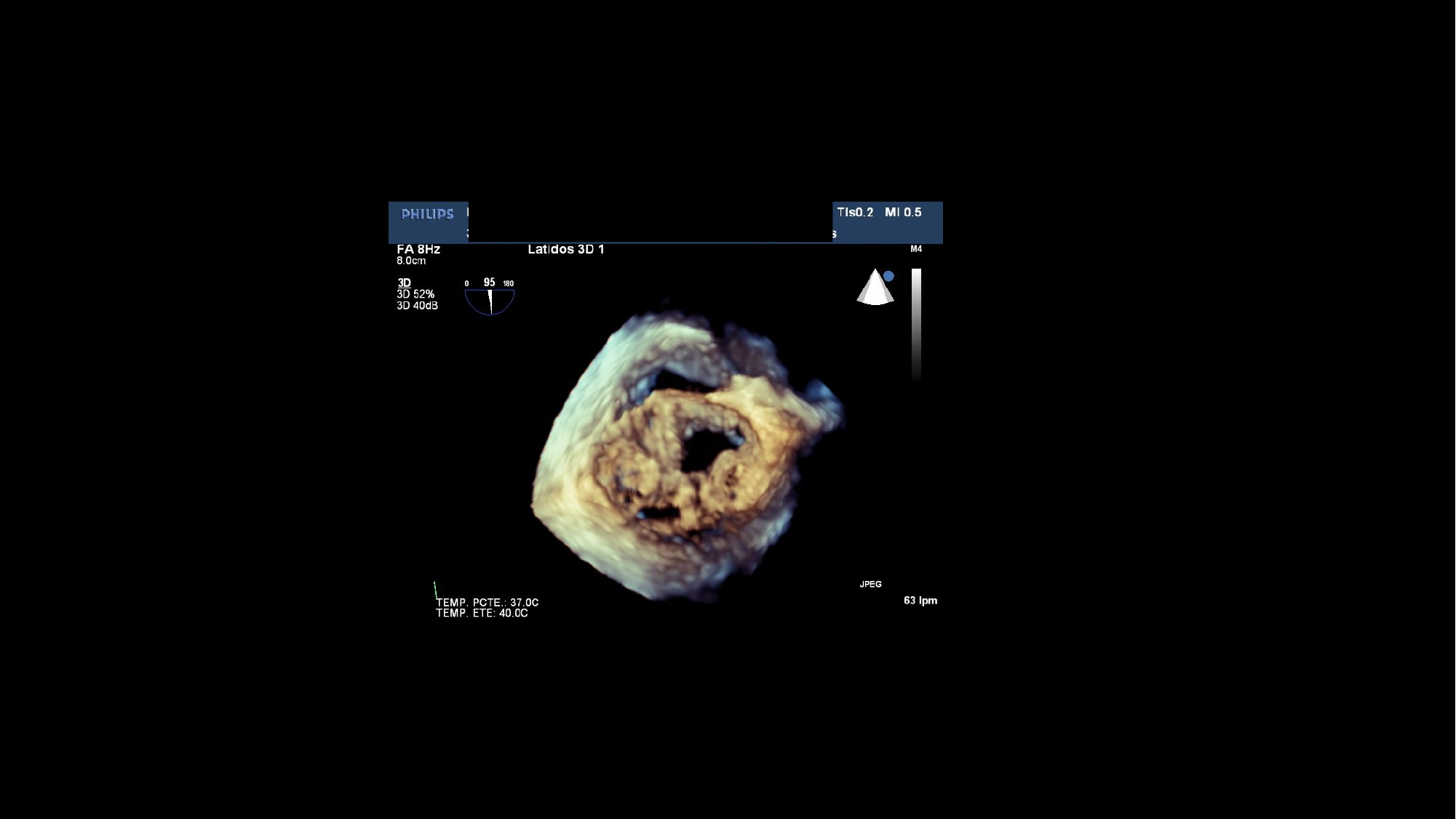

Supplement: Supplementary file 1 [file cells-15-01001-s001.zip › VIDEO S2.pptx]

## Slide 1
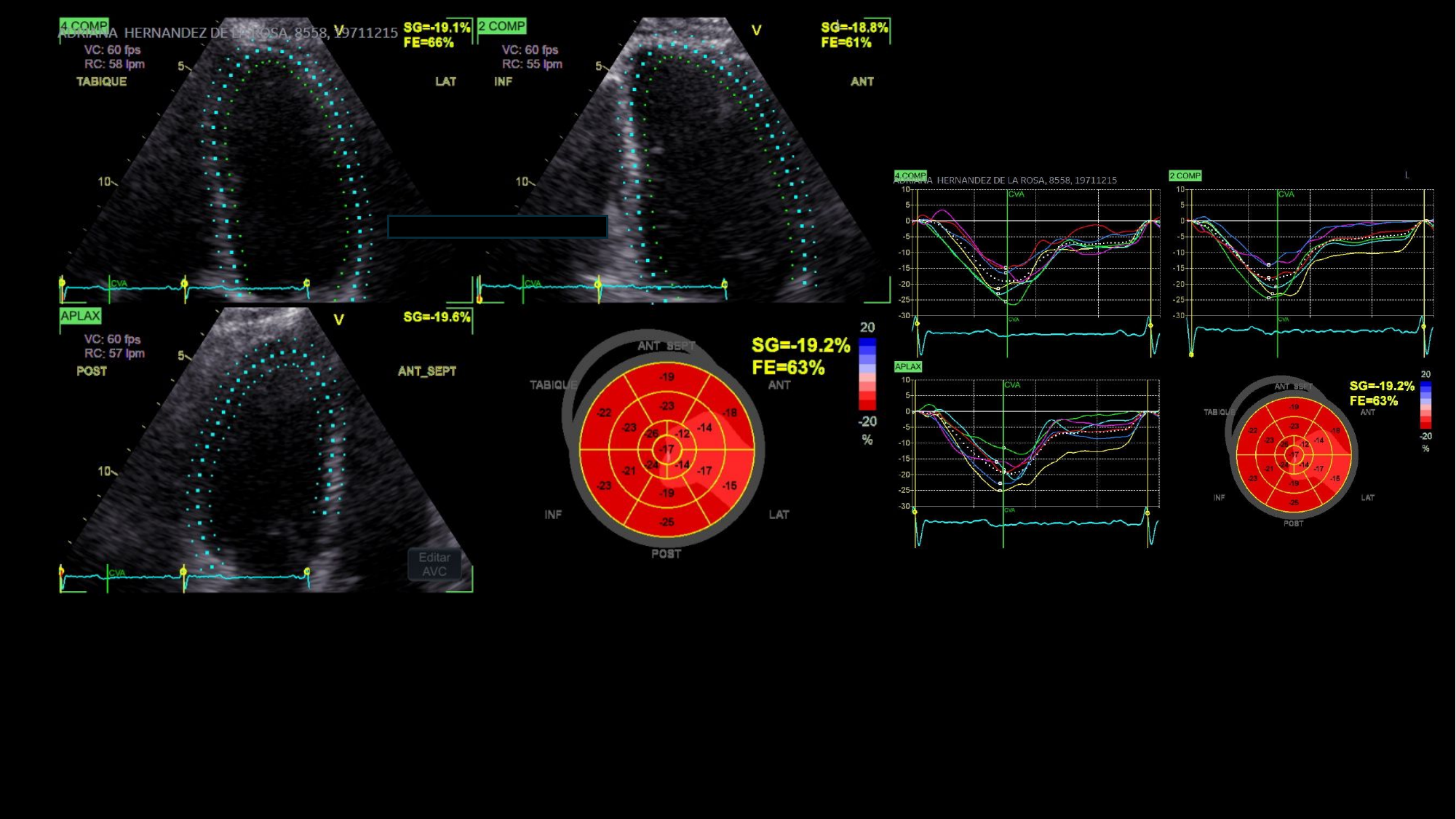

Supplement: Supplementary file 1 [file cells-15-01001-s001.zip › VIDEO S1.pptx]
